# Controls on Seasonal Atmosphere‐Ecosystem Carbon Dioxide Exchanges in a Temperate Salt Marsh

**DOI:** 10.1111/gcb.70740

**Published:** 2026-02-12

**Authors:** Jesus Ruiz‐Plancarte, Jose D. Fuentes, Karen J. McGlathery

**Affiliations:** ^1^ Department of Meteorology and Atmospheric Science The Pennsylvania State University University Park Pennsylvania USA; ^2^ Department of Meteorology Naval Postgraduate School Monterey California USA; ^3^ Department of Environmental Sciences University of Virginia Charlottesville Virginia USA

**Keywords:** carbon, coastal, inundation, salt marsh, tides, Virginia

## Abstract

Salt marshes play a vital role in the biogeochemistry of coastal zones, yet the biophysical controls on CO_2_ exchange with the atmosphere, or net ecosystem exchange (NEE, positive upwards) remain poorly quantified. We investigated a *Spartina alterniflora* monoculture salt marsh on the eastern shore of Virginia, United States, by estimating half‐hourly NEE from March 2016 to February 2017 using the eddy‐covariance method. Maximum marsh–atmosphere CO_2_ exchanges occurred during June and July when hourly averaged NEE values reached −10.0 ± 2.5 μmol CO_2_ m^−2^ s^−1^ (mean ±1 standard deviation). During the most productive time of the year, a tidal inundation of 0.7 m reduced daytime CO_2_ assimilation and nighttime CO_2_ release to the atmosphere by 5.0 ± 1.2 μmol CO_2_ m^−2^ s^−1^ and 3.0 ± 0.7 μmol CO_2_ m^−2^ s^−1^, respectively. Diffuse photosynthetically active radiation (PAR) conditions promoted quantum use efficiencies (*α*) of the ecosystem that were approximately three times greater than under direct PAR conditions (*α*_Cloudy_ = 0.012 ± 0.004 versus *α*_Clear_ = 0.004 ± 0.001 mol CO_2_ per (mol photons)). Under diffuse light, NEE increased more rapidly with PAR and photo‐saturation occurred at higher PAR levels compared to clear‐sky conditions. On average, under the influence of diffuse light, the assimilation of CO_2_ increased by 30% relative to equivalent PAR levels under direct sunlight. During March 2016 to February 2017 the marsh exchanged −269.1 ± 9.1 g of carbon per m^2^ with the atmosphere. The findings demonstrate that tides and light quality are key regulators of carbon cycling in tidal marshes. These factors should be incorporated into models of tidal marsh biogeochemistry, particularly as both are undergoing rapid changes due to sea level rise and atmospheric warming.

## Graphical abstract

We quantified net ecosystem exchange of carbon dioxide in a *Spartina alterniflora* salt marsh on the eastern shore of Virginia using eddy‐covariance measurements from March 2016 to February 2017. Peak carbon dioxide uptake occurred during summer, with tidal inundation reducing both daytime assimilation and nighttime respiration, while diffuse light conditions enhanced ecosystem quantum efficiency and increased carbon dioxide uptake by approximately 30% relative to direct sunlight. These findings identify tides and light quality as key regulators of marsh carbon cycling and highlight their importance for modeling coastal carbon dynamics under sea‐level rise and climate change.

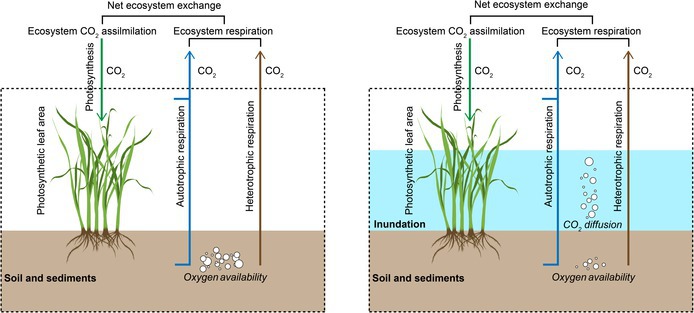

## 1 Introduction

Tidal wetlands reside at the interface between terrestrial and oceanic environments, thus making them highly susceptible to intense storms such as tropical and extra‐tropical cyclones, as well as to human encroachment. Wetlands are undergoing rapid changes in response to coastal engineering (Adam 2002) and sea‐level rise (Kirwan and Blum 2011). Despite occupying only 5%–8% of the Earth's surface, tidal wetlands store 83–233 Tg C year^−1^ (Chmura 2003; Cai 2011; Mcleod et al. 2011; Regnier et al. 2022), emphasizing their disproportionate role in global carbon sequestration. Sediment accretion is one key process driving carbon accumulation in tidal wetlands. In salt marshes along the eastern United States, sediment accretion rates average 2 mm year^−1^, while brackish and tidal freshwater marshes can accumulate sediments at rates 4–6 mm year^−1^ (Kastler and Wiberg 1996; Craft 2007; Loomis and Craft 2010). At the Virginia Coastal Reserve (VCR), Virginia, United States, organic accretion rates range from 5.9 to 6.4 mm year^−1^ (Kirwan and Blum 2011). Because salt marshes continually accrete and bury sediments, their rates of carbon sequestration per unit surface area are seven times greater than in forests (Chmura 2003). Both inorganic and organic matter contribute to vertical accretion, which enables marshes to store carbon at mean rates of 177 g C m^−2^ year^−1^ across mid‐Atlantic wetlands (Wang et al. 2019). Under stable environmental conditions, sediment accretion allows tidal marshes to keep pace with relative sea‐level rise (RSLR), which along the mid‐Atlantic coast of the United States averaged 2.0 ± 0.6 mm yr.^−1^ between 1950 and 2009 (Sallenger et al. 2012). However, where accretion cannot match the rate of RSLR, marshes may degrade into barren mudflats, causing a potential shift from a carbon sink to a carbon source (Kirwan and Mudd 2012; Macreadie et al. 2013).

Salt marsh vegetation contributes substantially to the net ecosystem carbon balance (Chapin et al. 2006; Najjar et al. 2018) owing to its inherently high rates of carbon dioxide (CO_2_) assimilation rates (e.g., Spartina leaves exhibit maximum carboxylation rates comparable to those of C3 crops, Kathilankal et al. (2011)). However, the net ecosystem CO_2_ exchange (NEE), which is the net flux from the ecosystem to the atmosphere, in tidally influenced marshes is governed not only by plant biomass, species composition, and phenology, but also by the depth, timing, and frequency of tidal inundation (Kathilankal et al. 2008). Because tidal flooding is a defining feature of coastal ecosystems, it is critical to quantify how inundation characteristics influence NEE, ecosystem respiration (RE), and gross primary production (GPP). Previous studies (Kathilankal et al. 2008; Artigas et al. 2015; Forbrich and Giblin 2015; Han et al. 2015) have shown that during the middle of the growing season, CO_2_ assimilation rates by the partly submerged plants decrease by as much as 60% due to inundation. Flooding of vegetated marsh surfaces reduces the amounts of photosynthetically active biomass exposed to the atmosphere and limits the diffusion of CO_2_ to carboxylation sites. Under flooding conditions, anaerobic (soil) and hypoxic (water column) environments often prevail, while part of the respired CO_2_ dissolves into the water column, resulting in suppressed ecosystem respiration (Schedlbauer et al. 2010; Koebsch et al. 2013).

Tidally influenced salt marshes are highly productive ecosystems, sustained by tidal oscillations that regularly deliver nutrient‐ and sediment‐rich waters (Fagherazzi et al. 2013; Czapla et al. 2020). Along the North American coast, their net primary productivity per unit area is about five times greater than that of estuaries and shelf waters (Najjar et al. 2018). The smooth cordgrass (S*partina*), which dominates the intertidal marshes, forms dense monocultures with leaf area indices (LAI) of 2.0–12.5 m^2^ m^−2^ (Schäfer et al. 2014; Drake et al. 2015). Its shoots alter water flows and trap suspended sediments, with sediment retention increasing with plant height and biomass (Morris et al. 2002; Mudd et al. 2010). As vegetation senesce, slow decomposition under anoxic conditions enriches soils with organic matter, while part of this material is exported during ebb tides (Kirwan et al. 2010; Guo et al. 2009; Cai 2011; Herrmann et al. 2015; Mitsch and Gosselink 2015). Strong feedbacks among tidal inundation, marsh vegetation, and sediment deposition enable marshes to remain effective carbon sink ecosystems (Fagherazzi and Priestas 2010; Fagherazzi et al. 2013; Mariotti and Carr 2014; Fitzgerald and Hughes 2019; Kirwan et al. 2023). With the accelerating RSLR, establishing baseline understanding of salt marsh function under varying levels of inundation is essential. Differences in CO_2_ uptake between flooded and non‐flooded conditions reflect the activity of photosynthetically exposed biomass, making marsh‐atmosphere CO_2_ fluxes a key metric of tidal influence on carbon exchange.

The quality of photosynthetically active radiation (PAR) controls the long‐term NEE of marsh ecosystems. In certain temperate regions the fraction of diffuse PAR is changing in response to variations in aerosol and cloudiness (Henderson‐Sellers 1989; Oliveira et al. 2011). Diffuse PAR often enhances quantum use efficiency relative to direct PAR because scattered photons can reach deeper into plant canopies, enabling lower leaves to receive light and contribute to carbon assimilation (Gu, Fuentes, et al. 1999; Knohl and Baldocchi 2008; Lee et al. 2018; Oliphant and Stoy 2018; Hemes et al. 2020). Furthermore, under the influence of diffuse light conditions, salt marshes exhibit substantially higher photosaturation thresholds compared to conditions dominated by direct sunlight (Kathilankal et al. 2008).

Temperature also influences NEE in tidal marshes, influencing both carbon assimilation in Spartina leaves (Kathilankal et al. 2011) through enzyme activation and the rate of ecosystem respiration. In temperate regions, respiration increases exponentially with soil temperature under non‐flooded conditions due to enhanced microbial activity (Kathilankal et al. 2008; Han et al. 2015). When air temperature drops below 283 K (10°C) during the growing season, daytime CO_2_ uptake declines (Teal and Howes 1996; Idaszkin and Bortolus 2011; Malone et al. 2016) as photosynthetic enzyme activity is suppressed. Under low‐tide conditions, NEE increases with temperature until reaching an optimum of about 303 K in Virginia marshes (Kathilankal et al. 2008), then decreases with further warming, likely due to photorespiration and photoinhibition (Farquhar et al. 1980). Given the projected temperature increases (Ji et al. 2014), it is essential to understand how warming alters NEE and carbon sequestration in salt marshes. *Spartina alterniflora* tolerates high salinity levels, up to approximately 35 parts per thousand (ppt; ≈0.6 M NaCl), with plant growth only moderately affected because the species can use sodium for osmotic adjustment in its shoots (Vasquez et al. 2006).

Because marsh–atmosphere CO_2_ exchange integrates ecosystem‐scale processes, this study addresses three related questions using full–growing‐season eddy‐covariance NEE. First, how does the salt‐marsh quantum use efficiency—the initial slope of the NEE–PAR response—differ under direct versus diffuse PAR? We hypothesize that, despite the marsh's low‐stature, sparse canopy (mean height 0.63±0.02 m; LAI 1.5±0.5 m^2^ m^−2^), diffuse light enhances photosynthetic efficiency by improving within‐canopy photon distribution. This effect is well documented for taller, denser plant canopies (Law et al. 2002; Gu et al. 2002; Wohlfahrt et al. 2008) but remains poorly characterized for salt marshes (Hawman et al. 2021), despite its importance for modeling GPP (Ryu et al. 2018; Yuan et al. 2014; Turner et al. 2006). Second, to what extent—and at which phenological stages—do tidally driven transient floods suppress NEE (day and night), as a function of inundation level and soil biogeochemical processes (Kathilankal et al. 2008; Forbrich and Giblin 2015; Knox et al. 2018; Nahrawi et al. 2020; Vázquez‐Lule and Vargas 2021)? Third, which environmental drivers (e.g., diffuse‐light fraction, temperature, VPD, water‐level metrics, and soil conditions) best explain intra‐ and inter‐seasonal variability in NEE, and what do these relationships imply for the marsh's annual CO_2_ balance as a net sink or source?

## 2 Methods

### 2.1 Site Characteristics

**FIGURE 1:**
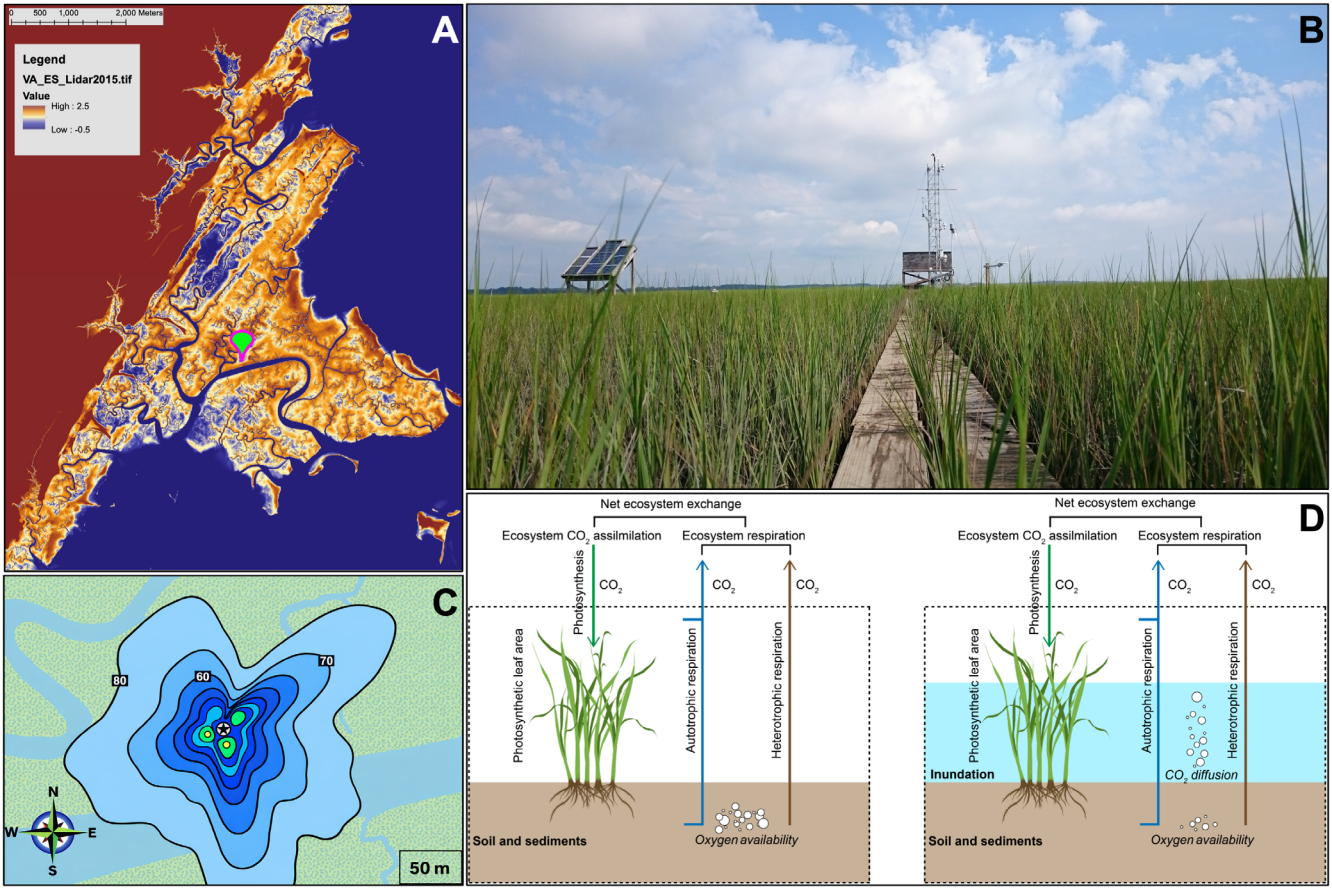
(A) Digital elevation map (meters) derived from LiDAR (Light Detection and Ranging) data for a marsh in Virginia, showing the location of the tower (green marker) and the surrounding topography (color shading). The map scale is shown in the upper left corner, and the elevation scale (from −0.5 to 2.5 m) is provided in the legend. (B) Picture of the 7‐m tower in the summer of 2016. (C) Flux footprint for the summer months during the daytime periods illustrating the contours (in %) for the areas where the CO_2_ fluxes emanated from (the star denotes location of the flux tower). (D) Conceptual illustration showing the processes dominating the CO_2_ exchange between the marsh and atmosphere when water is not present (left) and when the marsh is inundated (right).

**FIGURE 2:**
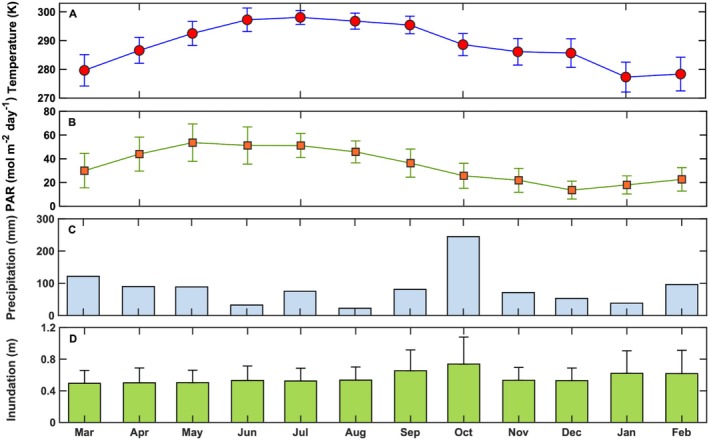
Seasonal patterns of (A) monthly average air temperature and standard deviation measured at 3.7 m above the ground, (B) the monthly mean PAR and standard deviation, (C) monthly total precipitation, and (D) averaged monthly inundation level and standard deviation from March 2016 to February 2017.

The study was conducted on a low marsh (latitude 37°24′39.78″N, longitude 75°49′59.63″W) of the Virginia Coast Reserve Long‐Term Ecological Research site (VCR‐LTER). The flux tower is located about 2 km from the mainland and about 85 m away from a major creek edge (Figure 1). The marsh experiences semi‐diurnal tidal inundation, with maximum water levels reaching up to 1.0 m above the mean soil surface. The highest inundation events typically occur from June to October, driven by warmer Atlantic Ocean and stormier weather patterns (Figure 2). Water salinity typically remains relatively stable year‐round, ranging from 28 to 30 Practical Salinity Units (PSU). The dominant vegetation is *Spartina alterniflora* , which is an intermediate C3‐C4 grass (Kathilankal et al. 2011), now reclassified as *Sporobolus alterniflora* (Peterson et al. 2014a, 2014b; Bortolus et al. 2019), and forms a continuous canopy across the site. The VCR‐LTER marsh is not fed by major rivers; instead, fresh water inputs derive primarily from precipitation and groundwater recharge. Consequently, nutrient loading to the lagoons is limited to atmospheric deposition and groundwater sources (McGlathery et al. 2007; Giordano et al. 2011). During the study period, June and August received little rainfall (< 20 mm), but October got almost 250 mm of rain due to extra‐tropical storms (Figure 2).

#### 2.1.1 Flux Tower Measurements

Continuous meteorological and eddy covariance measurements were made on a 7‐m flux tower located in the salt marsh (Figure 1). The eddy covariance unit was mounted at 3.7 m (= $z_{r}$) above the sediment surface. The eddy covariance system was comprised of a 3‐dimensional sonic anemometer (model R3‐50, Gill Instruments Limited, Lymington, Hampshire, UK) to measure air turbulent velocity (u = zonal component, v = meridional component, and w = vertical component) and an open‐path infrared gas analyzer (model LI‐7500, Licor Biosciences Inc., Lincoln, NE) to record water vapor and CO_2_ molar densities. Air turbulent velocity and CO_2_ molar density fluctuations were measured at the frequency of 20 Hz. Meteorological measurements defined the environmental conditions influencing the magnitude of fluxes and were recorded at 1 Hz, and the resulting data sets were stored on a data logger (model CR3000, Campbell Scientific Inc., Logan, UT). Meteorological measurements included air temperature (model 41342VC, RM Young, Traverse City, MI) at 2.1, 3.7 and 6.7 m above the surface and relative humidity (model 42342VC, Campbell Scientific Inc., Logan, UT) at 3.7 and 6.7 m. A propeller anemometer (model 05103, RM Young, Traverse City, MI) recorded wind speed and wind direction at the top of the tower. A tipping bucket (model TB4MM, Campbell Scientific Inc., Logan, UT), mounted at 5 m above the surface, registered rainfall amounts. A four‐component radiometer (model CNR4, Kipp & Zonen, Bohemia, NY), mounted at 2 m above the ground, measured incoming and outgoing solar and terrestrial radiation fluxes. Upward and downward facing quantum sensors (model PQS1, Kipp & Zonen, Bohemia, NY) on the same boom measured the incident and reflected PAR, respectively. Visible light measurements are used to determine direct and diffuse PAR (see Section 2.1.5). A pressure transducer (model CS541, Campbell Scientific Inc., Logan, UT) housed in a stainless‐steel case and placed in a well recorded water‐level fluctuations above the soil surface. Subsurface conditions characterized by three levels of soil temperature (model 105E, Campbell Scientific Inc., Logan, UT). Observations indicated that water salinity typically ranged between 28 and 30 PSU, consistent with long‐term measurements from nearby sites (Reagan et al. 2024). This variability reflects daily tidal flooding of the marsh, which lowers interstitial salinity through dilution and porewater exchange processes (Mitsch and Gosselink 2015). Table 1 summarizes the meteorological and ancillary measurements needed to interpret the NEE, and monthly mean environmental variables with standard deviations are shown in Figure 2.

**TABLE 1:** Summary of meteorological instrumentation and measurements collected at the Virginia Coast Reserve flux tower with their height in reference to soil surface.

Measurement	Height [m]	Instrument [model manufacturer]
Horizontal wind speed and direction	7.1	05103 RM Young
Precipitation	5.2	TB4MM Campbell Scientific
Air temperature and relative humidity	6.7, 2.1	42342VC Campbell Scientific
Air temperature	3.7	41342VC RM Young
Three‐dimensional wind velocity	3.7	R3 Gill Instruments
CO_2_ and H_2_O concentrations	3.7	LI‐7500 LICOR Biosciences
Incoming/outgoing short/long wave radiation	2.2	CNR4 Kipp & Zonen
Photosynthetically active radiation (PAR)	2.2	PSQ1 Kipp & Zonen
Soil heat flux	0.05	HFP01 Hukseflux
Inundation level	0	CS541 Campbell Scientific
Water temperature	0	CS541 Campbell Scientific

#### 2.1.2 Eddy Covariance Data Processing

The 20‐Hz eddy covariance data processing followed the protocol outlined in Fratini and Mauder (2014) to ensure consistency with previously published studies. Half‐hourly CO_2_ fluxes were calculated as the mean covariance between fluctuations of measured vertical velocity ($w^{\prime }$, m s^−1^) and CO_2_ mole fraction (μmol mol^−1^). The CO_2_ mole fractions were converted to molar density fluctuations ($\rho_{c}^{\prime }$, μmol CO_2_ m^−3^) using the ideal gas law. Fluxes were calculated assuming steady‐state conditions and horizontal homogeneity, and after rotating the coordinate system such that $\bar{v}$ and $\bar{w}$ are zero ($F_{{\mathrm{CO}}_{2}}=\bar{\rho_{c}^{\prime }w\prime }$) (Wilczak et al. 2001). The overbar denotes a 30‐min average, and the prime indicates the deviation from that mean. Data processing included spike removals (±4.5 standard deviations in a window of 15 values, with an exception to when four consecutive values fit the criterion, then values were not labeled as spikes) as explained in Vickers and Mahrt (1997) to ensure the necessary statistical quality of the raw time series to compute fluxes. The double coordinate rotation (Tanner and Thurtell 1969) eliminated errors associated with misalignments of the sonic anemometer. To eliminate an overestimation in the calculated $F_{{\mathrm{CO}}_{2}}$ caused by air density fluctuations, data were corrected using established methodologies (Webb et al. 1980). The contribution of CO_2_ storage ($F_{s,{\mathrm{CO}}_{2}}=\int_{0}^{z_{r}}\frac{{\partial \rho }_{c}}{\partial t}\mathrm{dz}$) to the $F_{{\mathrm{CO}}_{2}}$ in the air column below the eddy covariance unit was not considered because the air column below the sensor was too shallow to make a significant contribution. To ensure that the estimated $F_{{\mathrm{CO}}_{2}}$ applied to the salt marsh, the flux footprint was estimated following the method described in Kljun et al. (2015). The footprint calculation required the input of eddy covariance measurement height ($z_{r}$), friction velocity (u_*_), turbulence statistics such as the standard deviation of v (*σ*_v_), wind speed at $z_{r}$ ($\bar{u}\left(z_{r}\right)$), roughness length for the momentum sink (z_0_), wind direction, and Obukhov length ($L=-\frac{u_{*}^{3}\;\bar{T_{V}}}{k\;g\;\left(\bar{w^{\prime }\;\theta_{V}^{\prime }}\right)}$; $\bar{T_{V}}$ is the average virtual temperature, *k* is the von Kármán's constant (0.4), and $\left(\bar{w^{\prime }\;\theta_{V}^{\prime }}\right)$ is the kinematic virtual heat flux). Figure 1C provides the results for the seasonal footprint analyses. Eddy covariance data gaps resulted because of power outages, instrument malfunction, out of range readings from the sonic anemometer or the gas analyzer due to precipitation, and inadequate flux footprint (which encompassed a creek located 85–95 m south of the flux tower, Figure 1C). As a result, approximately 29% of the 1‐year record was excluded from the analysis. Data gaps varied by variable: missing meteorological measurements accounted for ~5% of the record (filled using nearby VCR‐LTER weather stations), missing 3‐D sonic anemometer and CO_2_ flux data accounted for ~29%, and missing inundation data accounted for ~10% (filled using the nearest VCR‐LTER tide station). The largest of these values (29%) was therefore reported as the overall fraction of missing data for flux calculations. For the seasonal salt marsh‐atmosphere CO_2_ exchange, data gaps were filled using information from two nearby meteorological towers, that provided air temperature (Tair), wind speed, PAR, vapor pressure deficit (VPD), and inundation levels, maintained by the VCR‐LTER staff. The $F_{{\mathrm{CO}}_{2}}$ gaps were then filled using an artificial neural network approach (Knox et al. 2018). The corrected and quality‐controlled $F_{{\mathrm{CO}}_{2}}$ data are hereafter described as the NEE, with seasonal mean values reported along with their standard deviations.

#### 2.1.3 Seasonal Plant Phenology

Amounts and age of photosynthetically active biomass strongly influence marsh CO_2_ assimilation throughout the growing season. To identify plant growth stages and determine the drivers of seasonal NEE variability, we combined measurements of harvested biomass (ratio of live to dead) with PAR trends to delineate transitions between growing to non‐growing periods. Aboveground biomass was sampled monthly in six replicate quadrants (0.25 m × 0.25 m), following the methods of Morris et al. (1990). To minimize disturbance, quadrants were located outside the flux tower footprint in areas representative of the surrounding vegetation. In the laboratory, living and dead plant material were separated, and plant height, number of leaves per plant, and length of individual leaves were measured. Samples were weighed fresh, then oven dried at 60°C for 48 h. Specific leaf area (SLA) was calculated, and LAI for each month (mean ± standard deviation) was derived as the product of SLA and biomass per unit ground. Aboveground net primary production (ANPP) was estimated using the Smalley (1960) method, which sums the monthly changes in live and dead biomass.

#### 2.1.4 Determination of Quantum Use Efficiency

One objective of this study was to quantify the salt marsh quantum use efficiency (*α* = mol CO_2_ m^−2^ s^−1^ per (mol photons m^−2^ s^−1^)). Quantum efficiency was derived by fitting PAR to daytime NEE obtained during low‐tide conditions (inundation level < 0.02 m). For each month, half‐hourly PAR and NEE data were fitted to the Michaelis–Menten relationship (Ruimy et al. 1995):

(1) $${\mathrm{NEE}}_{\mathrm{day}}=-\frac{A_{\max}\;\alpha \;\mathrm{PAR}}{A_{\max}+\alpha \;\mathrm{PAR}}+{\mathrm{RE}}_{\mathrm{day}}$$

where *A*_max_ (μmol CO_2_ m^−2^ s^−1^) is the maximum NEE under saturating PAR, and RE_day_ (μmol CO_2_ m^−2^ s^−1^) represents daytime ecosystem respiration. The unknowns in Equation (1) were obtained using MATLAB's nonlinear regression solver (*fitnlm*, version 2017b, Mathworks Inc., Natick, MA), with initial values of *A*_max_ = 10 μmol CO_2_ m^−2^ s^−1^, *α* = 0.005 mol CO_2_ m^−2^ s^−1^ per (mol photons m^−2^ s^−1^), and RE_day_ = 1 μmol CO_2_ m^−2^ s^−1^. The solver iteratively calculated the coefficient's means and standard deviations, and all fits were statistically significant (*p*‐values < 0.001). The *V*_*cmax*_ was estimated using coupled stomatal conductance and canopy energy balance equations based on the Farquhar C_4_ photosynthesis model (Farquhar et al. 1980; Chen et al. 1994):

(2) $$V_{c\;\max}=\frac{V_{c\;\max}\;\exp\left[\left(-\frac{E\;}{T}\;\left(\frac{1}{T_{L}}-\frac{1}{T_{25}}\right)\right)\right]\times \left[1+\exp\left(\frac{S\;T_{25}-H}{R\;T_{25}}\right)\right]\;}{\left[1+\exp\left(\frac{S\;T_{L}-H}{R\;T}\right)\right]}$$

Regression coefficients for Spartina were adopted from Kathilankal et al. (2011): V_*c*25_ = 44.25 *μ*mol CO_2_ m^−2^ s^−1^, E = 43113.9 J mol^−1^, *R* = 8.314 J mol^−1^ K^−1^ (universal gas constant), T_25_ is the reference temperature of 298 K, S = 3533.2 J mol^−1^ K^−1^, and H = 1116346.2 J mol^−1^. Canopy temperature (T_*L*_) was calculated using the surface energy balance (neglecting heat storage and metabolic heat production) and the linearization of the Penman (1948) evapotranspiration formulation (Campbell and Norman 1998, eq. 14.6): $T_{L}=T_{\mathrm{air}}+\frac{\gamma^{*}}{s+\gamma^{*}\;}\;\left[\frac{R_{\mathrm{abs}}-\epsilon \;\sigma \;T_{a}^{4}}{g_{\mathrm{hr}}\;c_{p}}-\frac{V\;P\;D}{P_{a}\;\gamma^{*}}\right]$ where R_*abs*_ = R_*net*_ + *εσ*T^4^_*air*_ in W m^−2^, s (= ∆*/P*_*a*_) is the slope of the saturation vapor pressure curve, and *γ⁎* = γ gₕᵣ/gᵥ is the apparent psychrometric constant. Conductances were defined as *g*_*hr*_ = *g*_*ha*_ + *g*_*r*_, where $g_{\mathrm{ha}}=1.4\times 0.135\;\sqrt{\frac{U}{d}}$, *g*_*r*_ = 4*εσ*T^3^_*a*_ */c*_*p*_, and $g_{v}=1.4\times 0.147\;\sqrt{\frac{U}{d}}$, wit d = 0.7 × leaf width (3 cm). Constants included ε = 0.9, *σ* = 5.67 × 10^−8^ W m^−2^ K^−4^, *cₚ* = 29.3 J mol^−1^ K^−1^, and γ = 6.66 × 10^−4^ °C^−1^. The slope of the vapor pressure function (Δ) was calculated as.

Δ = [4217/(Tₐ + 240.97)^2^] × 611 exp.[(17.50 Tₐ)/(Tₐ + 240.97)] (Pa °C^−1^). Calculations required as input air temperature (T_*a*_, K), vapor pressure deficit (VPD, Pa), net radiation (R_*net*_, W m^−2^), air pressure (P_*a*_, Pa), and wind speed (U, m s^−1^) from half hourly averages.

#### 2.1.5 Influences of Direct and Diffuse Light on Net Ecosystem Exchange

Estimates of diffuse PAR (PAR_f_) were required to evaluate the effects of light quality on CO_2_ assimilation by the salt marsh. The methodology followed Appendix A of Gu et al. (2002) and is summarized here for completeness. Using data restricted to low‐tide conditions (58% of NEE data), half‐hourly PAR_f_ was estimated from ambient temperature (T_a_), relative humidity (RH), solar zenith angle (β, degrees), total incoming solar irradiance (S_t_), and top‐of‐atmosphere the solar irradiance (S_e_) for a plane parallel to the Earth's surface (Spitters et al. 1986). The S_e_ values were derived from day of year, solar zenith and elevation angles, and eq. A2 in Gu et al. (2002). The total diffuse irradiance (S_f_) fraction of S_e_ (S_f_/S_e_) was calculated from the clearness index (k_t_ = S_t_/S_e_) using empirical relationships for distinct k_t_ ranges (Reindl et al. 1990). Following Alados and Alados‐Arboledas (1999) and Perez et al. (1990), diffuse PAR was then estimated from S_f_, dew point temperature (T_d_), sky brightness (δ = S_f_/S_e_), and sky clearness (i.e., ε = [1 + (S_t_ − S_f_)/(S_f_ cosβ) + 1.041β^3^]/[1 + 1.041β^3^]). Direct PAR (PAR_d_) was obtained as PAR_d_ = PAR − PAR_f_. These relationships have been extensively validated in prior studies (Gu, Fuentes, et al. 1999; Gu et al. 2002; Oliphant and Stoy 2018). The resulting PAR_d_ and PAR_f_ values were incorporated into Equation (1) to solve for the non‐linear regression coefficients (*A*_max_, *α*, and RE_day_), quantifying the effects of light quality on CO_2_ assimilation. Coefficients and their standard deviation were estimated as in Section 2.1. To examine the influence of PAR_d_ on NEE, PAR values were grouped into 150 μmol m^−2^ s^−1^ bins. Across the full study period, approximately 49% of the half‐hourly PAR data were classified as diffuse conditions using the approach outline above.

#### 2.1.6 Estimating Inundation Influences on Daytime Net Ecosystem Exchange

The ecosystem‐level response to tidal inundation, when the plant canopy was either partially or fully submerged, was assessed by combining the average plant height (0.63 ± 0.02 m) with daily tidal amplitudes reaching up to 1.0 m. Flooding frequency (defined as inundation levels > 0.02 m) was quantified from half‐hourly records across the diurnal cycle. Using the hyperbolic light‐response model Equation (1) to estimate NEE in the absence of inundation effects (NEE_L_), daytime changes in NEE due to flooding (∆NEE_day_) were calculated as the difference between NEE and NEE_L_ (∆NEE_day_ = NEE − NEE_L_). The ∆NEE_day_ values, including both cloudy and sunny periods, were grouped into 5‐cm inundation intervals to quantify the net change in CO_2_ exchange as a function of water depth. Because daytime NEE is typically negative (indicating CO_2_ uptake), inundation generally decreases its magnitude, producing positive ∆NEE_day_ values. Standard deviations were reported for each inundation class.

#### 2.1.7 Nighttime Carbon Dioxide Flux

In coastal ecosystems, nighttime NEE is primarily modulated by soil temperature and degree of tidal inundation. To investigate these effects, nighttime NEE values were fitted to an Arrhenius‐type function for two regimes: low (water level < 0.02 m) and high (water level > 0.5 m). Nighttime was defined as periods when incoming solar irradiance was < 10 W m^−2^; when irradiance data were unavailable, the onset of nighttime was estimated from latitude and longitude using the formulation of Spitters et al. (1986). The dependence of nighttime NEE on soil temperature was determined using Lloyd and Taylor (1994):

(3) $$\mathrm{NEE}={\mathrm{NEE}}_{\mathrm{ref}}\;\exp\left[E_{0}\;\left(\frac{1}{T_{\mathrm{ref}}-T_{0}}-\frac{1}{T_{s}-T_{0}}\right)\right]$$

where NEE_ref_ (μmol CO_2_ m^−2^ s^−1^) is the nighttime NEE at a reference temperature (T_ref_, which has the value of 283 K; 10°C), E_0_ (K) is the temperature‐dependent activation energy sensitivity, T_s_ (K) is the absolute soil temperature measured at 5 cm depth, and T_0_ is a prescribed temperature (224 K, Lloyd and Taylor (1994)). Previous studies (e.g., Reichstein et al. 2005; Moffat et al. 2007) demonstrated that a 15‐day window provides a sufficient temperature range for reliable nonlinear regression while minimizing seasonal variability in NEE. Nighttime NEE data were partitioned into low‐ and high‐tide conditions, and regression coefficients (e.g., NEE_ref_, E_0_) were obtained for each 15‐day period using Matlab's non‐linear optimization solver *fmincon* (version 2017b, Mathworks Inc., Natick, MA). Optimization employed lower and upper bounds of (10, 500) and (0, 240), respectively, and initial values of (1, 350) for NEE_ref_ (μmol CO_2_ m^−2^ s^−1^) and E_0_ (K). Values of E_0_ remained mostly constant (= 240 K) across seasons and inundation conditions, except during autumn (= 247 K) and winter (= 252 K) under low‐tide conditions. In contrast, NEE_ref_ varied markedly with inundation: low tide in spring = 1.12, summer = 1.06, autumn = 1.01, winter = 1.06, annual = 0.99; high tide in spring = 0.27, summer = 0.43, autumn = 0.36, winter = 0.81 (all in μmol CO_2_ m^−2^ s^−1^).

## 3 Results

### 3.1 Environmental Conditions Influencing Net Ecosystem Exchange

**FIGURE 3:**
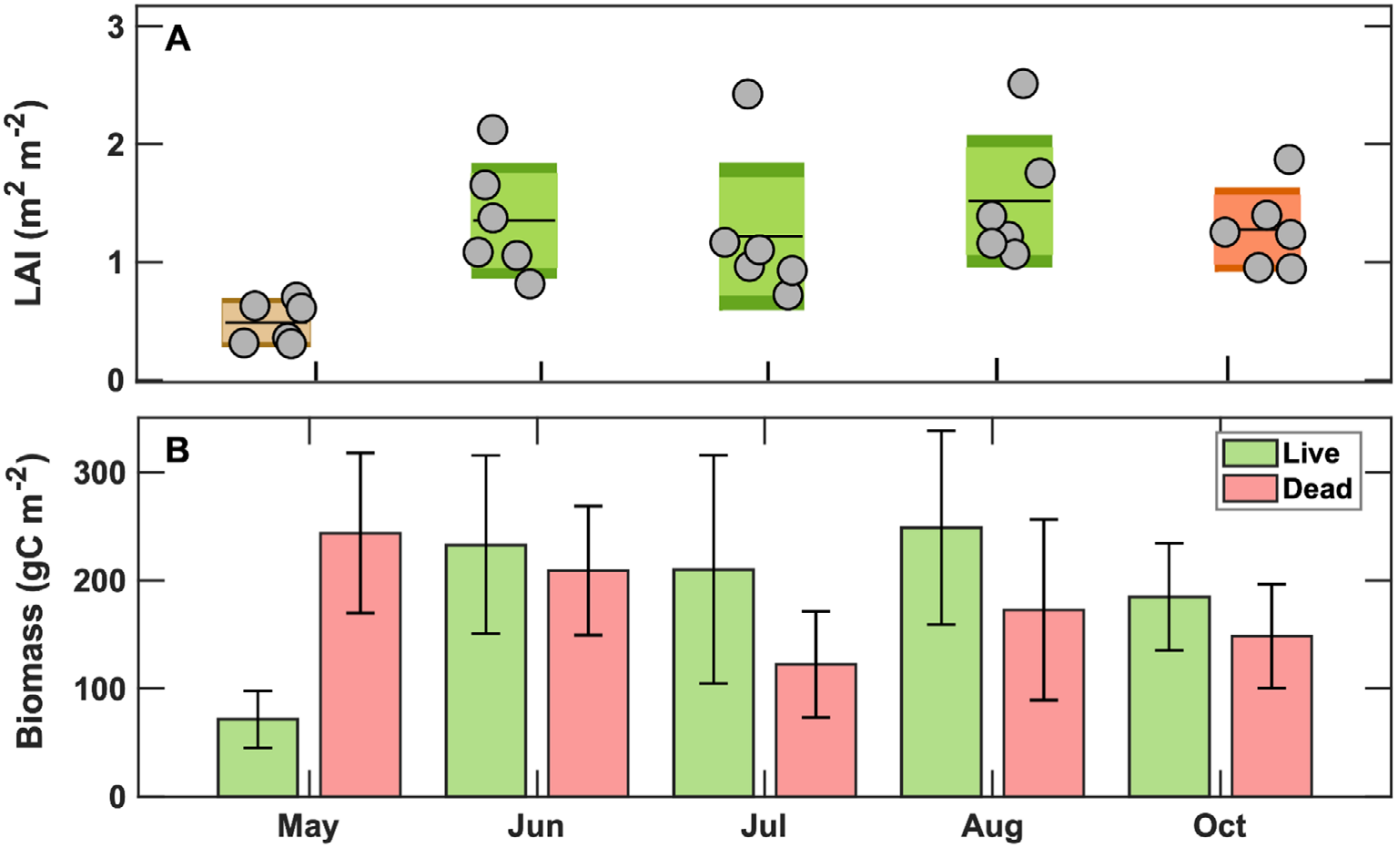
(A) Mean leaf area index (LAI) is shown with a black line, 95% of the samples lie within the lighter shade, the standard deviation is shown in darker shade, and individual measurements are shown as dots. (B) Monthly total Spartina live and dead biomass with standard deviation values after drying in 60°C oven for 48 h.

During 2016 the VCR‐LTER salt marsh monthly averaged incoming PAR and air temperature reached maximum values of 51 ± 10 mol m^2^ day^−1^ and 298 ± 2 K, respectively, in July (Figure 2a,b). The salt marsh growing season commenced in April, when the monthly averaged air temperature exceeded 286 ± 5 K and PAR reached 44 ± 14 mol m^−2^ day^−1^ (Figure 2c). Fresh water inputs to the marsh via precipitation occurred throughout all the months of the year, with October being the wettest month with total rainfall of about 244 mm due to tropical storm Matthew. Two semidiurnal tides caused flooding of the salt marsh, with water levels markedly higher between June and October, with averaged monthly water levels exceeding 0.52 ± 0.18 m above the soil surface (Figure 2d). The maximum monthly averaged inundation level of 0.74 ± 0.34 m occurred in October 2016. In the spring, when air temperatures ranged from 279 ± 6 to 292 ± 4 K, Spartina leaf emergence took place. By May, mean LAI reached 0.5 ± 0.2 m^2^ m^−2^ (Figure 3a). As leaves continued to develop and the canopy reached maturity, the LAI attained the maximum value 1.5 ± 0.5 m^2^ m^−2^ in August. Plant canopy development and leaf senescence (Figure 3a) closely tracked the seasonal patterns of PAR (Figure 2a). Leaf senescence started in September and the physiologically active biomass reached lower values (184 ± 49 g C m^−2^) in October compared to the ones (248 ± 89 g C m^−2^) experienced in August (Figure 3b). Over the 2016 growing season, aboveground physiologically active biomass ranged from 71 ± 26 g C m^−2^ in May to 248 ± 89 g C m^−2^ in August.

Plant canopy reached an average height of 0.63 ± 0.02 m, with LAI exhibiting strong seasonal variability, averaging 1.5 ± 0.3 and peaking at 2.4 m^2^ m^−2^ (Figure 3a). Monthly biomass sampling distinguished live and dead aboveground material, showing live biomass increasing from 71.4 ± 23.4 g C m^−2^ early in the season (May) to 229.9 ± 97.4 g C m^−2^ at peak growth (July–August). Summing live biomass across the growing season yielded an aboveground ANPP of 250.5 ± 70.6 g C m^−2^ (Figure 3b).

#### 3.1.1 Seasonal Net Ecosystem Exchange and Physiological Attributes

**FIGURE 4:**
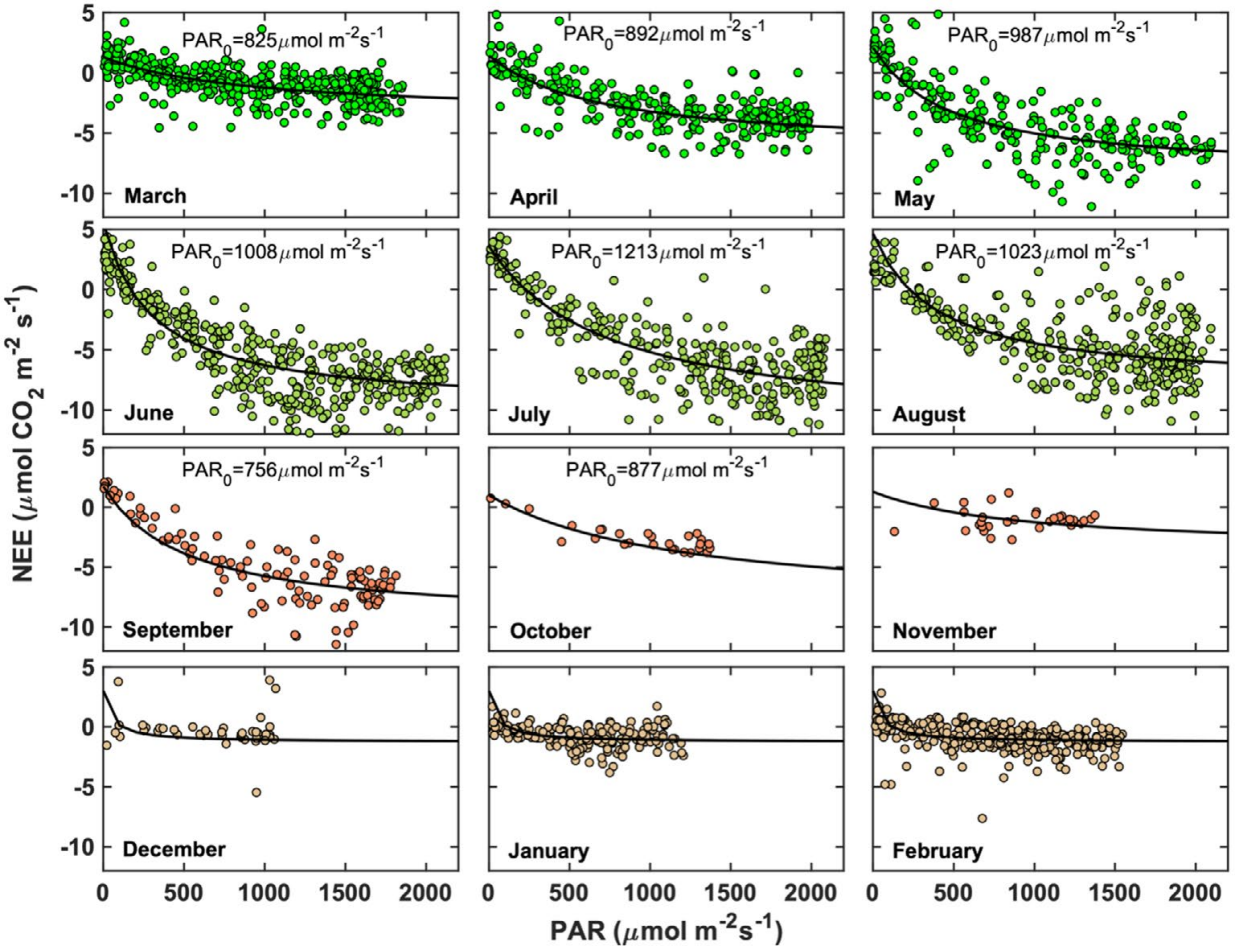
Half‐hourly net ecosystem CO_2_ exchange as a function of PAR during daytime and low tide (inundation level < 0.02 m) conditions from March 2016 to February 2017. The PAR‐NEE model fit Equation (1) is shown in black. The maximum light saturating value (PAR_0_) is shown for the months during the growing season.

Strong seasonal dynamics in environmental drivers (Figure 2) and plant phenology (Figure 3) produced pronounced variations in NEE throughout the growing season. Following leaf emergence in late March and early April, NEE exhibited a characteristic response to PAR (Figure 4) during low‐tide conditions (inundation < 0.02 m). Within monthly segregated data, the NEE‐PAR relationship showed substantial variability. Early in the growing season (e.g., April), maximum CO_2_ assimilation reached −5.0 ± 1.6 μmol CO_2_ m^−2^ s^−1^ at a photosaturation level of 890 ± 24 μmol photon m^−2^ s^−1^. As the season progressed, NEE increased in magnitude, attaining mean values of −8.0 ± 4.7 μmol CO_2_ m^−2^ s^−1^ during June—July, coinciding with optimal environmental conditions (Figure 2) and greatest amounts of photosynthetically active biomass (Figure 3). Seasonal photosaturation levels increased from approximately 825 ± 24 μmol photon m^−2^ s^−1^ in early spring to about 1213 ± 22 μmol photon m^−2^ s^−1^ by mid‐summer (Figure 4), consistent with values reported for other salt marshes (Lee et al. 2015; Han et al. 2015; Zhong et al. 2016). As foliage senescence commenced in September and October, NEE declined steadily, and by November the marsh ceased functioning as a net CO_2_ sink. From November through February, NEE remained near 0.0 ± 1.7 μmol CO_2_ m^−2^ s^−1^ due to the scarcity of photosynthetically active biomass. Although Figure 4 presents daytime NEE, positive NEE values occasionally occurred under low‐light conditions (PAR < 100 μmol photon m^−2^ s^−1^), averaging 5 ± 1.8 μmol CO_2_ m^−2^ s^−1^ during June—August. These values likely reflect residual canopy and soil respiration and CO_2_ transport from the canopy to the atmosphere during the night‐to‐day‐transition (Kathilankal et al. 2008; Moffett et al. 2010; Knox et al. 2018). At night and shortly after sunrise, vertical CO_2_ gradients commonly develop over vegetated landscapes, with concentrations decreasing with height due to the canopy acting as a dominant CO_2_ source from soil and plant respiration (Gu, Shugart, et al. 1999).

**FIGURE 5:**
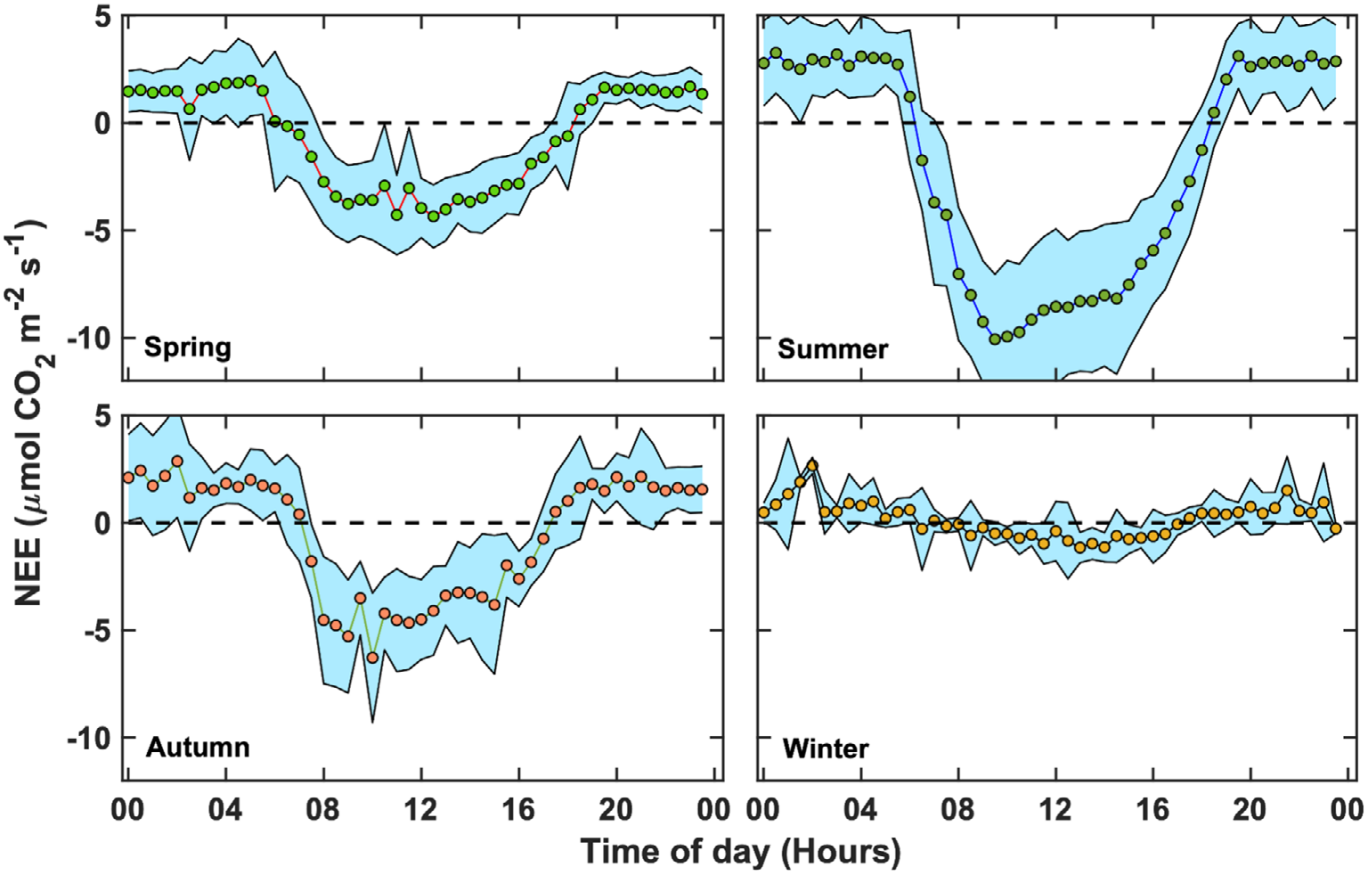
Seasonally averaged daily cycles of net ecosystem CO_2_ exchange during low tide (inundation level < 0.02 m conditions) for spring (March–May), summer (June–August), autumn (September–November), and winter (December–February). Shaded areas represent the standard deviation around the mean values, which are represented with circles.

Marked seasonal patterns in the diel cycles of averaged NEE reflected variations in environmental drivers (e.g., PAR, air temperature, and soil temperature) and the amounts of photosynthetically active biomass that supported CO_2_ assimilation at sub‐hourly scales (Figure 5). On average, the salt marsh functioned as a CO_2_ sink from 1 to 2 h after sunrise until sunset. Maximum marsh−atmosphere CO_2_ exchanges occurred between 10:00 to 16:00 local time, when hourly averaged NEE reached −5.0 ± 1.5 μmol CO_2_ m^−2^ s^−1^ in spring and −10.0 ± 2.5 μmol CO_2_ m^−2^ s^−1^ in summer (Figure 5a,b), coinciding with peak air temperature, PAR, and atmospheric turbulence (data not shown). By early autumn, NEE declined, with mid‐day NEE decreasing to −5.0 ± 1.9 μmol CO_2_ m^−2^ s^−1^ (Figure 5c), consistent with lead senescence (Figure 3) and concurrent declines in PAR and temperature (Figure 2). One recurrent feature in NEE time series was the mid‐morning (9:30 to 12:00 h) reduction in CO_2_ uptake observed later in the season (Figure 5). During these times, the ecosystem routinely experienced reductions in stomatal conductance to CO_2_ diffusion into the leaves (Barr et al. 2009; Kathilankal et al. 2008) as a consequence of the excessive evaporative demand (data not shown), evidently observed when VPD > 15 hPa (Kathilankal et al. 2008). Similar diel NEE patterns have been reported for other wetland ecosystems (Barr et al. 2009; Lee et al. 2015; Knox et al. 2018). Nighttime NEE increased as the growing season progressed, reaching maximum of 5 ± 1.8 μmol CO_2_ m^−2^ s^−1^ during June–August. Nighttime CO_2_ exchange started to rapidly decrease in September, reflecting plant phenological stage and decreasing temperatures (< 285 K). During the winter (December, January, and February), the marsh became a weak sink of CO_2_, with a mean nighttime NEE near 0 ± 0.87 μmol CO_2_ m^−2^ s^−1^ (Figure 5).

**FIGURE 6:**
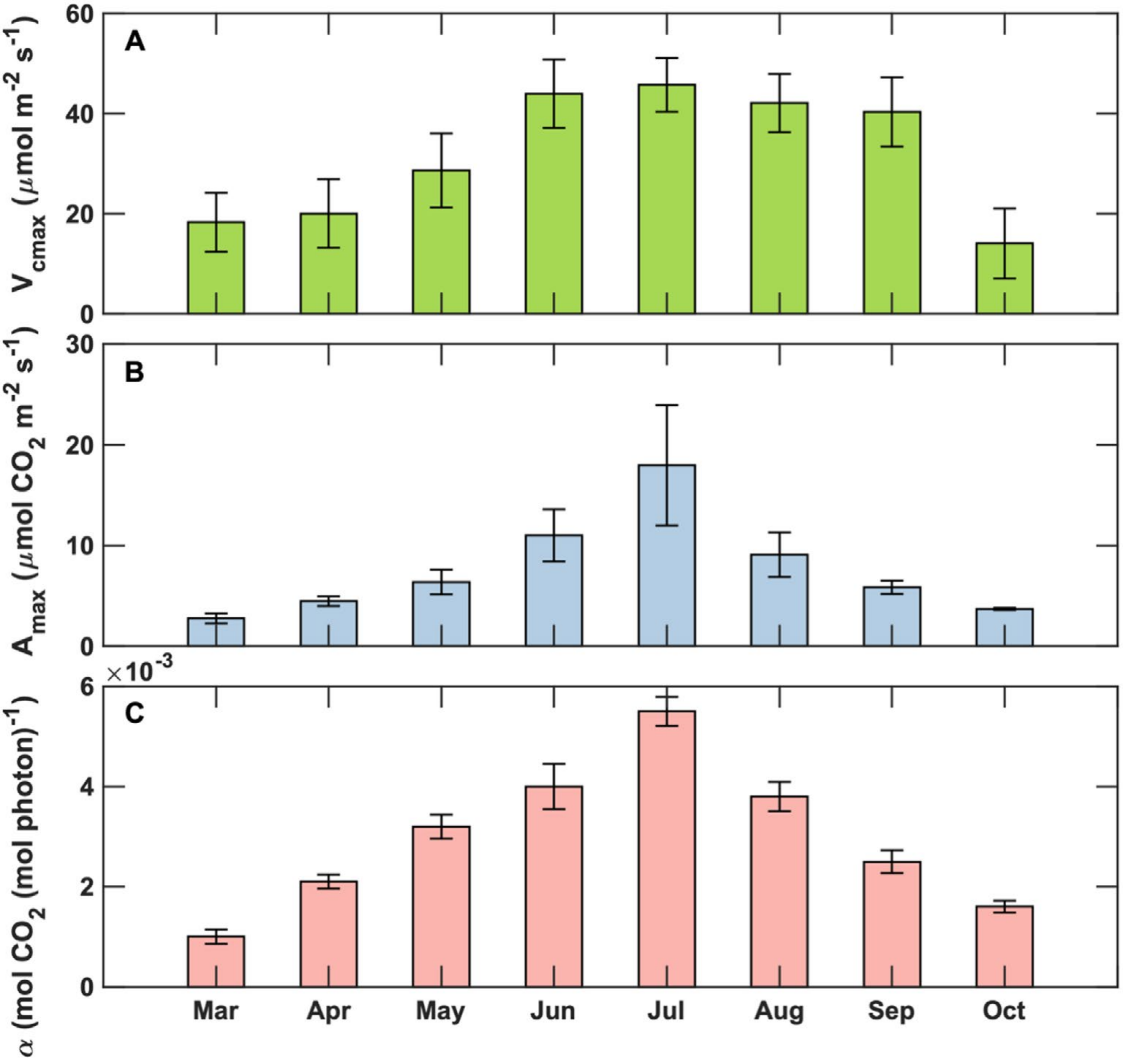
Estimated monthly averages of (A) maximum ecosystem rate of carboxylation (*V*_cmax_), (B) maximum net ecosystem CO_2_ exchange at saturating PAR conditions (*A*_max_), and (C) quantum yields (*α*) during low‐tide conditions from March to October 2016. Error bars denote standard deviation values.

Ecosystem physiological attributes exhibited marked seasonal variability throughout the growing season. Under low‐tide conditions, averaged *V*_cmax_ increased from 19.1 ± 6.2 μmol CO_2_ m^−2^ s^−1^ in Mar—April to a seasonal maximum of 45.7 ± 5.3 μmol CO_2_ m^−2^ s^−1^ in July (Figure 6a). *A*_max_ similarly followed a seasonal trend, rising from 3.6 ± 0.5 in March–April to 17.6 ± 5.9 μmol CO_2_ m^−2^ s^−1^ in July. Highest values of *α* occurred in July, with monthly averaged values of 0.0055 ± 0.0003 mol CO_2_ (mol photon)^−1^. Seasonal changes in *V*_cmax_, *A*_max_, and *α* closely paralleled shifts in vegetation phenology (Figure 3) and in environmental drivers such as temperature and PAR (Figure 2). Summertime canopy‐scale values of *V*_cmax_, *A*_max_, and *α* (40 < *V*_cmax_ < 60 μmol CO_2_ m^−2^ s^−1^, 9 < *A*_max_ < 18 μmol CO_2_ m^−2^ s^−1^, 3 × 10^−3^ < *α* < 5 × 10^−3^ mol CO_2_ (mol photons)^−1^) were consistent with previously reported Spartina leaf‐level measurements under foliage temperatures of 300–315 K (Kathilankal et al. 2011). These seasonal physiological characteristics (Figure 6) provide essential inputs for biospheric and biogeochemical models (e.g., Kirwan and Mudd 2012) aimed at quantifying carbon cycling in tidal marsh ecosystems.

**FIGURE 7:**
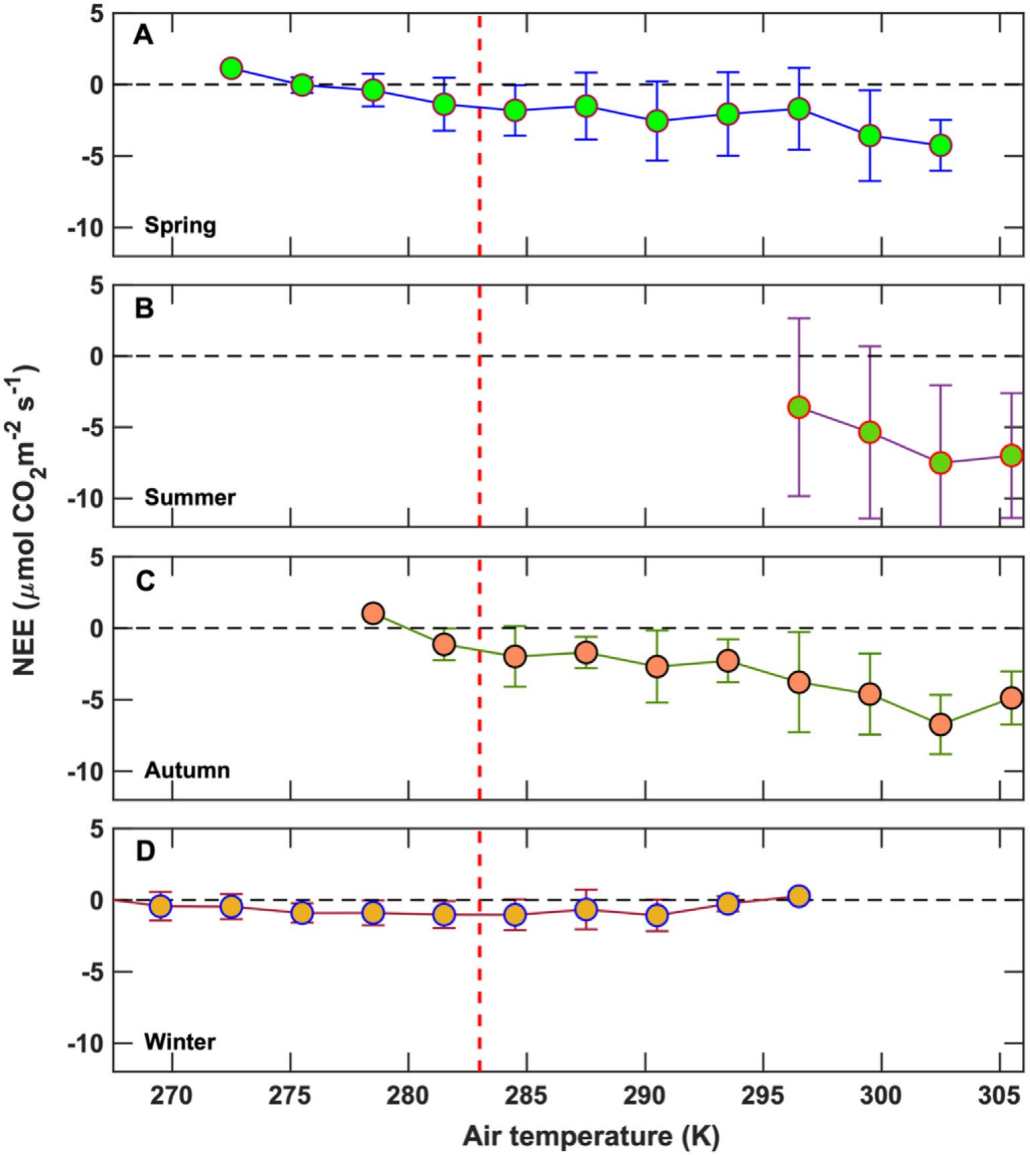
The relationship between net ecosystem CO_2_ exchange and air temperature during daytime hours and low‐tide conditions for (A) spring (March–May), (B) summer (June–August), (C) autumn (September–November), and (D) winter (December–February). The bars indicate the standard deviation. The red dashed line is the literature value (283 K; 10°C) for decreased CO_2_ uptake by vegetation.

Air temperature is a key variable regulating NEE on both seasonal and annual timescales (Baldocchi 2008). Rising temperatures enhance ecosystem respiration, increasing CO_2_ release to the atmosphere, while concurrently stimulating photosynthetic carboxylation rates. Under daytime and low‐tide conditions, net carbon uptake by the marsh increased with temperature until reaching an optimum near 303 K, beyond which NEE plateaued during the summer (Figure 7). In contrast, during autumn, when temperatures exceeded 303 K, NEE declined with additional warming (Figure 7). Comparable temperature responses have been observed previously at this site (Kathilankal et al. 2011). More broadly, long‐term warming trends are known to amplify carbon losses from northern ecosystems during spring and autumn (Piao et al. 2008).

**FIGURE 8:**
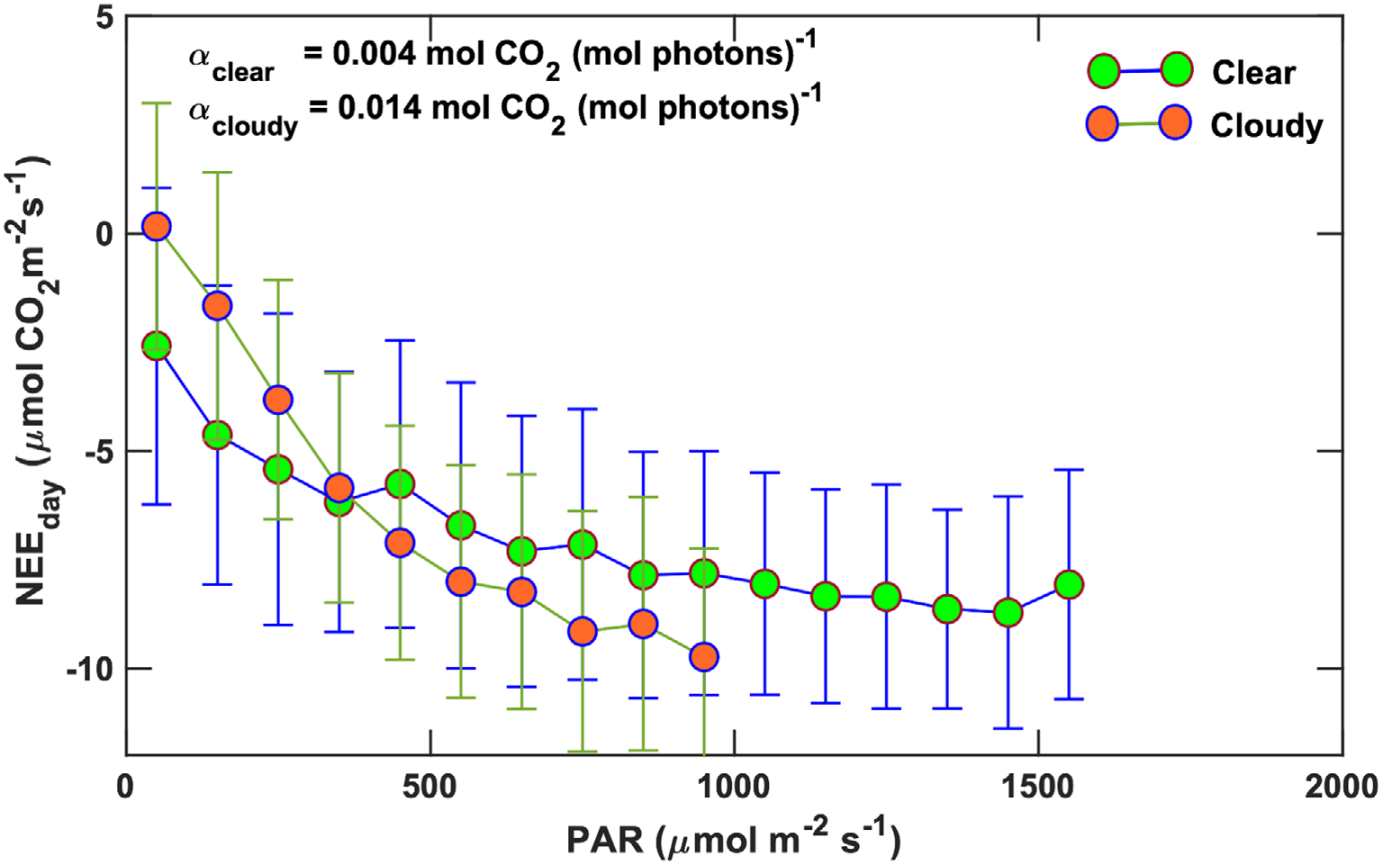
Relationship between daytime net ecosystem CO_2_ exchange and photosynthetically active radiation during cloudy and clear conditions. The error bars indicate the standard deviation. The Michaelis–Menten function of Equation (1) was best fit to each set of binned values to obtain the values of the quantum use efficiency (*α*) for cloudy and clear conditions.

**FIGURE 9:**
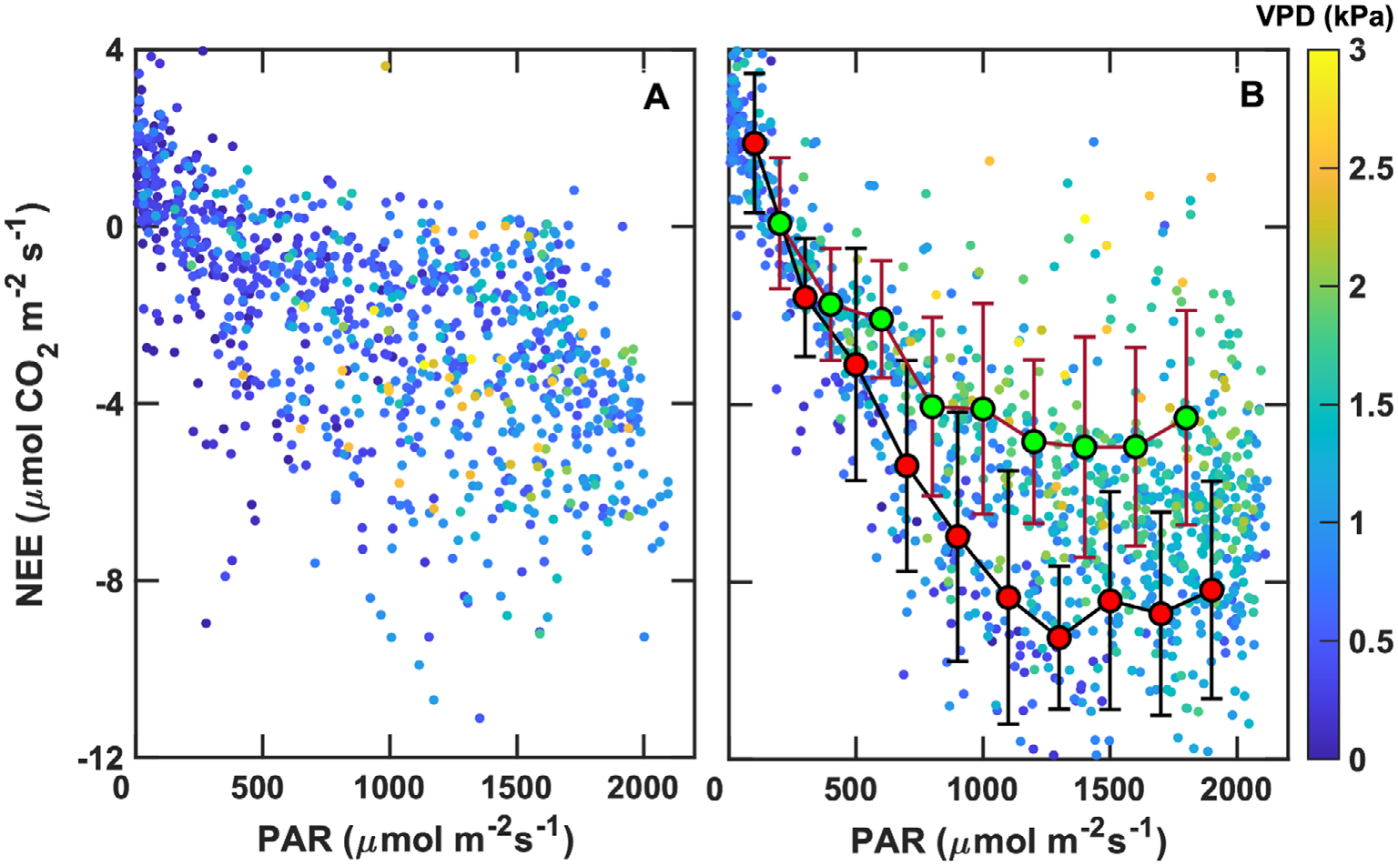
Daytime net ecosystem exchange (NEE) as a function of photosynthetically active radiation (PAR) during (A) spring and (B) summer periods, with vapor pressure deficit (VPD) indicated by the color scale (see color bar). For the summer data, separate response curves were fitted to subsets of observations characterized by high VPD (> 2 kPa; gold line with green circles) and low VPD (< 1 kPa; black line with red circles) to illustrate the influence of atmospheric moisture demand on NEE under comparable PAR conditions. In contrast, no discernible relationship between NEE and PAR could be identified for the spring dataset.

The salt marsh exhibited higher quantum use efficiency under cloudy, diffuse light conditions compared to clear‐sky periods. At a PAR level of 800 μmol photon m^−2^ s^−1^, average NEE was −5.5 ± 0.4 μmol CO_2_ m^−2^ s^−1^ under cloudless conditions, whereas it increased in magnitude to −8.0 ± 0.5 μmol CO_2_ m^−2^ s^−1^ under diffuse light (Figure 8). Enhanced CO_2_ assimilation during cloudy periods can be ascribed to the *cloud‐gap effect* (Gu, Shugart, et al. 1999; Lee et al. 2018; Oliphant and Stoy 2018), in which vegetation receives greater diffuse irradiance, along with comparable total PAR, due to enhanced reflection and scattering of sunlight by clouds and aerosols. Diffuse light is particularly common in coastal ecosystems because of frequent cloud cover and aerosols formed from salt spray. Previous studies (Roderick et al. 2001; Gu et al. 2002) have shown that terrestrial ecosystems assimilate more CO_2_ under hazy conditions. For deciduous forests in North America, the NEE enhancement can be 30%–60% greater under cloudy skies (Gu, Fuentes, et al. 1999). Although the salt marsh in 2016 had a short canopy (h_c_ = 0.63 ± 0.02 m) and an average maximum LAI of 1.5 ± 0.3, the Spartina vegetation experienced approximately a 30% enhancement in NEE (Figure 8) under cloudy conditions. This response in NEE due to the quality of light aligns with previous findings (Kathilankal et al. 2008), which reported 25%–50% greater quantum use efficiency (*α*) under diffuse compared to direct light. When comparing NEE versus PAR relationships (Figure 9), the reduced CO_2_ assimilation under clear skies is consistent with higher VPDs that limit stomatal conductance (Lasslop et al. 2010; Knox et al. 2018). In addition, for the VCR LTER marsh, increased NEE during cloudy conditions may also reflect the indirect effects of lower thermal and radiation stress (Kathilankal et al. 2008).

#### 3.1.2 Controls on Nighttime Net Ecosystem Exchange

**FIGURE 10:**
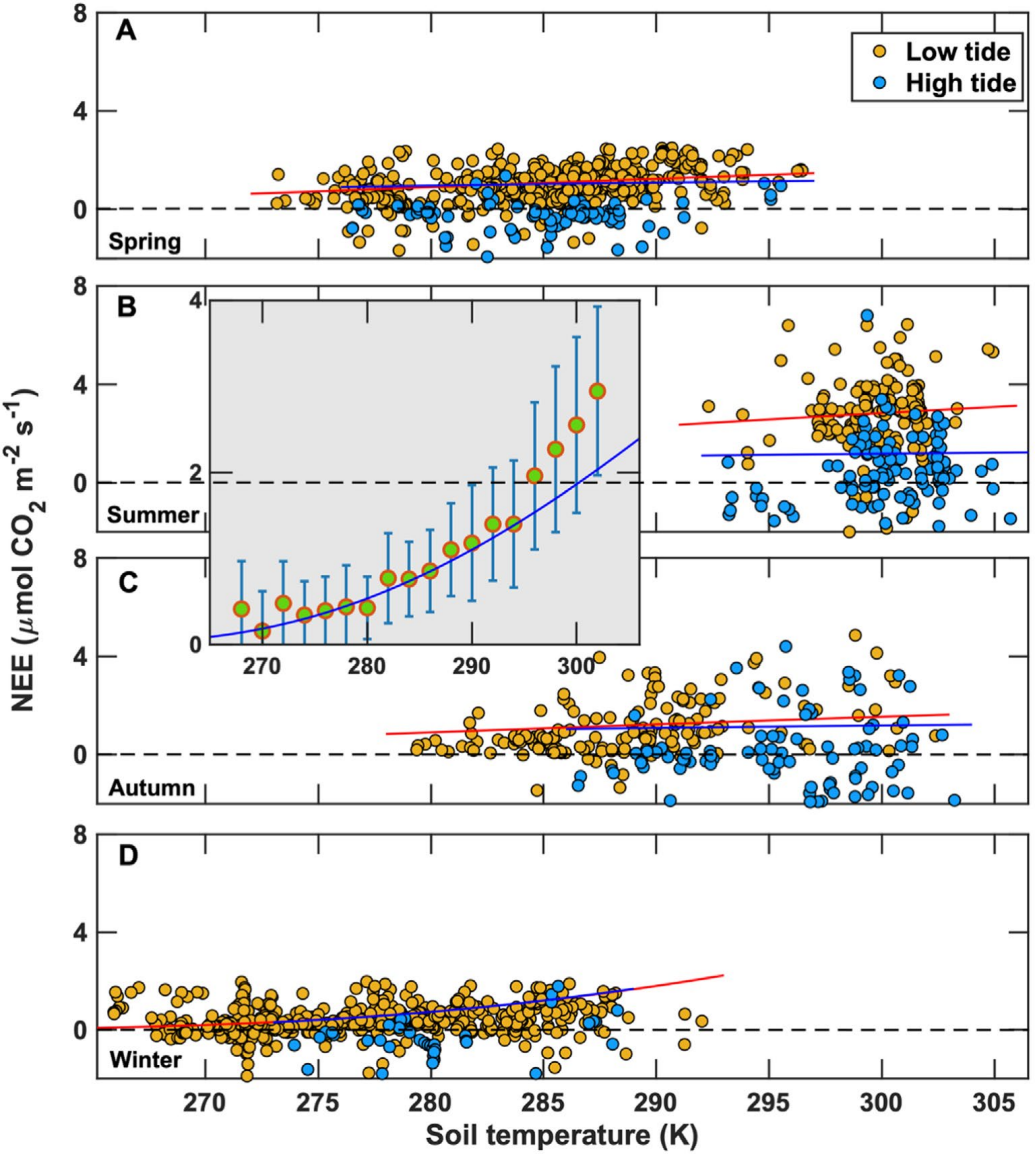
Relationship between soil temperature, measured at 5 cm depth, and nighttime net ecosystem CO_2_ exchange for (A) spring (March–May), (B) summer (June–August), (C) autumn (September–November), and (D) winter (December–February) during low‐ and high‐tide conditions. Lines represent the best fit relationship, and their coefficients are summarized in Table 2. The inset provides a summary of the nighttime net ecosystem CO_2_ exchange for low‐tide conditions for the full measurement period (March 2016 to February 2017).

Respiration is a key process regulating the net carbon exchange in salt marsh ecosystems. Its magnitude is primarily controlled by plant biomass, soil temperature, and inundation levels (Figure 10). During low tides, nighttime NEE is interpreted as ecosystem respiration and typically increases exponentially with soil temperature, consistent with Equation (2) and with observations in terrestrial ecosystems (Chen and Tian 2005; Peng et al. 2009; Wu et al. 2011). In contrast, high‐tide conditions substantially suppress nighttime NEE. When analyzed for the summer and fall seasons (Figure 10), nighttime NEE under high tides exhibited a linear relationship with soil temperature (*R* ^2^ = 0.364), reaching 6.0 ± 0.17 μmol CO_2_ m^−2^ s^−1^ at 303 K, compared to 2.0 ± 0.13 μmol CO_2_ m^−2^ s^−1^ during low tides. Under low tide conditions, respiration followed the Arrhenius function (Equation (2), see inset in Figure 10), with activation energies (*E*_a_) varying seasonally (Table 2). Reduced nighttime NEE during flooding likely reflects limited CO_2_ diffusion through the water column and reduced oxygen availability for both heterotrophic and autotrophic respiration. Maximum nighttime NEE occurred between June and August, when respiration ranged from 4 to 7 μmol CO_2_ m^−2^ s^−1^, whereas minimum values of 1–3 μmol CO_2_ m^−2^ s^−1^ were observed between December and February (Figure 10). These results are consistent with previous findings for other wetlands (mangrove forests: Barr et al. (2010); Troxler et al. 2015; salt marsh: Guo et al. 2009; Moffett et al. 2010; Artigas et al. 2015; Forbrich and Giblin 2015; Han et al. 2015; Wang et al. 2016; Knox et al. 2018).

**TABLE 2:** Results from the regression analyses for nighttime carbon dioxide exchange quantities included in Equation (2) by season and inundation level. Low corresponds to exposed soil conditions while high equates to inundated conditions. The annual values correspond to the inset of Figure 11. Units of NEE and NEE_ref_ (shown with standard deviation) are in μmol CO_2_ m^−2^ s^−1^, and units of T_s_ and E_0_ are in *K*.

	Spring		Summer		Autumn		Winter		Annual
	Low	High	Low	High	Low	High	Low	High	Low
Mean NEE	1.08	0.05	2.39	0.81	0.82	0.07	0.40	0.10	1.17
Mean T_s_	286.4	286.1	299.8	300.3	289.7	291.8	279.2	278.4	290.9
NEE_ref_	0.93 ± 0.20	0.29 ± 0.42	1.05 ± 0.19	0.44 ± 0.18	0.67 ± 0.11	0.35 ± 0.06	0.56 ± 0.18	0.43 ± 0.35	0.78 ± 0.195
E_0_	240	240	240	240	240	240	240	240	240
RMSE	0.42	0.47	1.11	1.33	0.98	1.30	0.43	0.44	0.69

#### 3.1.3 Tidal Influence on Net Ecosystem Exchange

**FIGURE 11:**
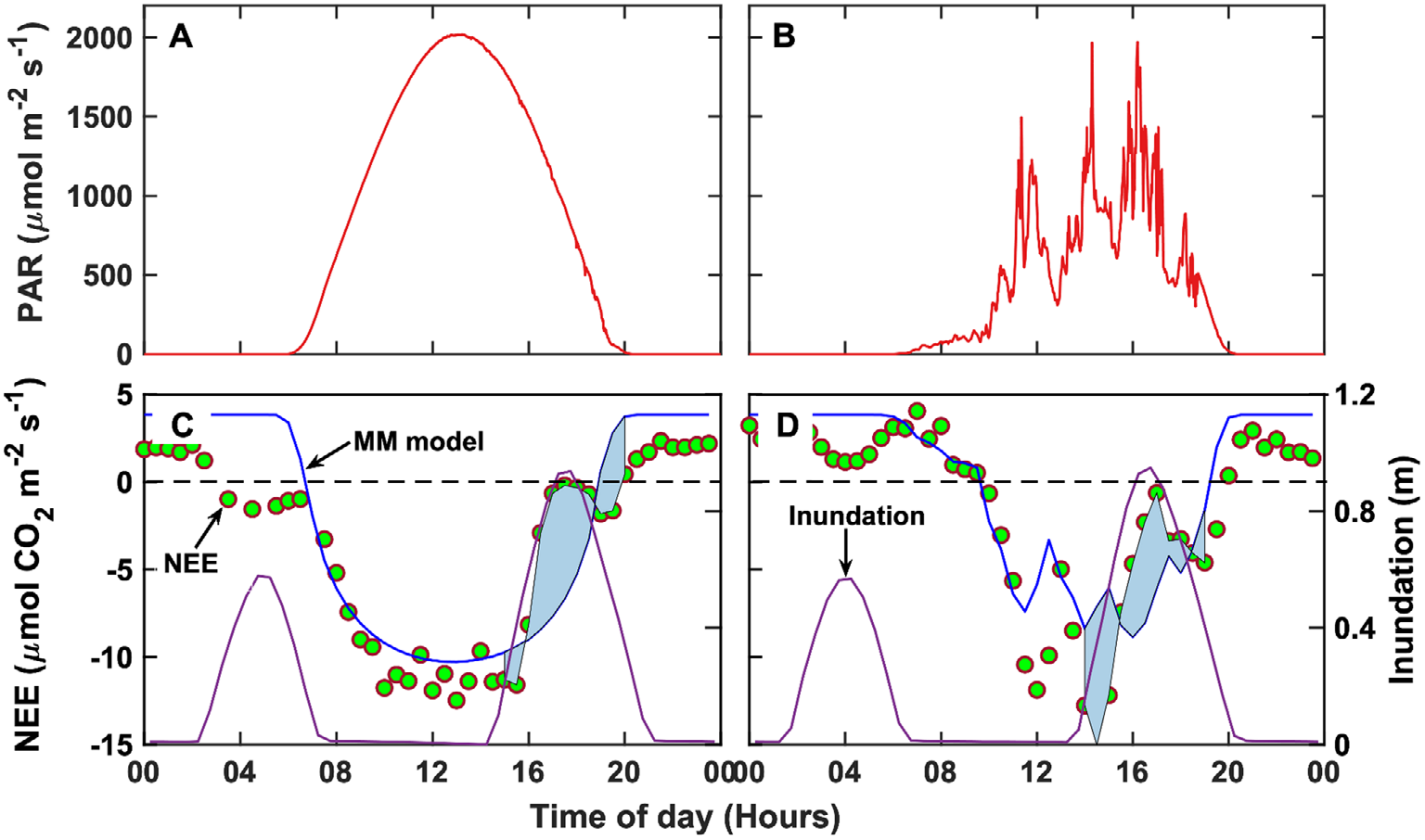
Diel cycles of photosynthetically active radiation for (A) a clear day and (B) a mostly cloudy day and corresponding net ecosystem CO_2_ exchange and inundation level for (C) the clear day and (D) the mostly cloudy day. The difference between the Michaelis–Menten fit (solid blue) and eddy covariance NEE (green circles) on both days (C, D) indicates the change in NEE (shaded region) resulting due to tidal inundation.

Duration, time of day, and level of inundation modulated the magnitude of the NEE diel cycles for the salt marsh. The short canopy (0.63 ± 0.02 m. Figure 3), coupled with a maximum tidal amplitude of approximately 1.0 m (Figure 2), provided ideal conditions for quantifying ecosystem‐level responses to inundation. Example days under cloudless and mostly cloudy conditions (Figure 11) both showed reductions in nighttime CO_2_ release and daytime CO_2_ assimilation. Nighttime tides reaching about 0.6 m reduced NEE from 3 to almost 0 μmol CO_2_ m^−2^ s^−1^, likely due to suppressed respiration and restricted gas diffusion through the water column. Under flooded conditions, limited oxygen diffusion constrained both autotrophic and heterotrophic respiration. The overlying water column also created a physical barrier to CO_2_ efflux, while oxygen availability produced hypoxic or anoxic conditions that further reduced respiration (McNicol and Silver 2014). On the cloudless day (Figure 11a), high tide between 15:30 and 20:00 h produced 1.0 m of inundation, which caused full submergence of the vegetation, reducing NEE to −0.6 μmol CO_2_ m^−2^ s^−1^ compared to a modeled non‐inundated NEE_L_ of −8.0 μmol CO_2_ m^−2^ s^−1^, which yielded an NEE reduction of 7.3 μmol CO_2_ m^−2^ s^−1^. On the cloudy day (Figure 11b), variable PAR and a similar inundation level of 1.0 m (∼16:30 h) reduced NEE to −3.0 μmol CO_2_ m^−2^ s^−1^ versus a modeled NEE_L_ of −8.7 μmol CO_2_ m^−2^ s^−1^, yielding a 5.7 μmol CO_2_ m^−2^ s^−1^ decrease. Flooding reduced effective photosynthetic leaf area as shoot and leaves became partially or completely submerged. Reduced CO_2_ diffusion and diminished light transfer in the turbid water column further restricted photosynthesis (Kathilankal et al. 2011; Colmer et al. 2011). In general, NEE declined with the rising tides (Figure 11) due to decreased active biomass and hindered water‐atmosphere CO_2_ exchange.

**FIGURE 12:**
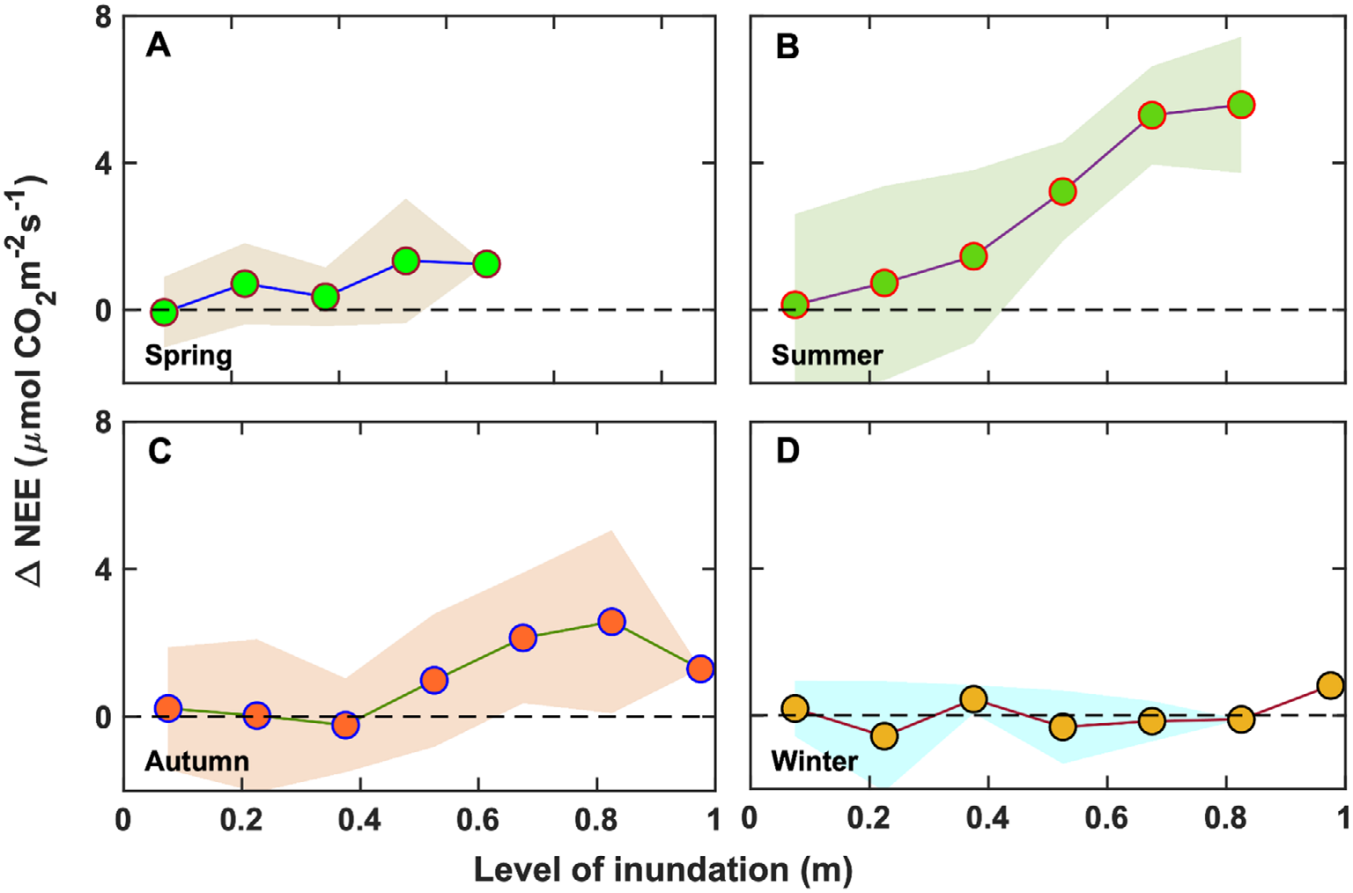
Reductions in daytime net ecosystem CO_2_ exchange (∆NEE_day_) calculated for (A) spring (March–May), (B) summer (June–August), (C) autumn (September–November), and (D) winter (December–February) as a function of inundation level. Circles represent average ∆NEE_day_ values and the shaded region represents the standard deviation.

Seasonal changes in salt marsh NEE due to flooding reflected variations in canopy development and the amount of physiologically active biomass. When integrated over 0.05‐m inundation intervals, ∆NEE_day_ values revealed clear seasonal patterns in CO_2_ assimilation losses during flooding. In the spring, when the canopy was still developing (Figure 3), maximum ∆NEE_day_ values were less than 3 μmol CO_2_ m^−2^ s^−1^ (Figure 12a). As the canopy matured in summer, ∆NEE_day_ increased exponentially with inundation depth, reaching maximum average reductions of 6.0 ± 0.8 μmol CO_2_ m^−2^ s^−1^ (Figure 12b). During autumn, when plants began to senesce, ∆NEE_day_ remained below 4.0 ± 0.6 μmol CO_2_ m^−2^ s^−1^. The larger standard deviations observed in summer and autumn likely reflected variability in PAR, temperature, and the fraction of submerged active biomass affecting CO_2_ exchange. In winter, inundation had no measurable influence on ∆NEE_day_ as its averaged value was 0 μmol CO_2_ m^−2^ s^−1^. Comparable studies (Forbrich and Giblin 2015) have reported smaller NEE reductions (~5%) for tall marshes in New England under the influence of bi‐weekly tides. For short‐stature marshes flooded twice a day by tides, the NEE reductions ranged from 30% to 60% (Kathilankal et al. 2008; Han et al. 2015). The present results (Figure 11) confirm that inundation depth, duration, frequency, and the amount of submerged physiologically active biomass directly modulate marsh CO_2_ exchange, collectively diminishing the ecosystem's carbon sink capacity (Kathilankal et al. 2008; Guo et al. 2009; Moffett et al. 2010; Artigas et al. 2015; Forbrich and Giblin 2015; Han et al. 2015; Knox et al. 2018). Continued long‐term measurements will be essential to assess the cumulative impacts of relative sea‐level rise and storm‐driven flooding, particularly where marsh migration inland is constrained (Kirwan and Megonigal 2013).

#### 3.1.4 Annual Net Ecosystem Exchange

**FIGURE 13:**
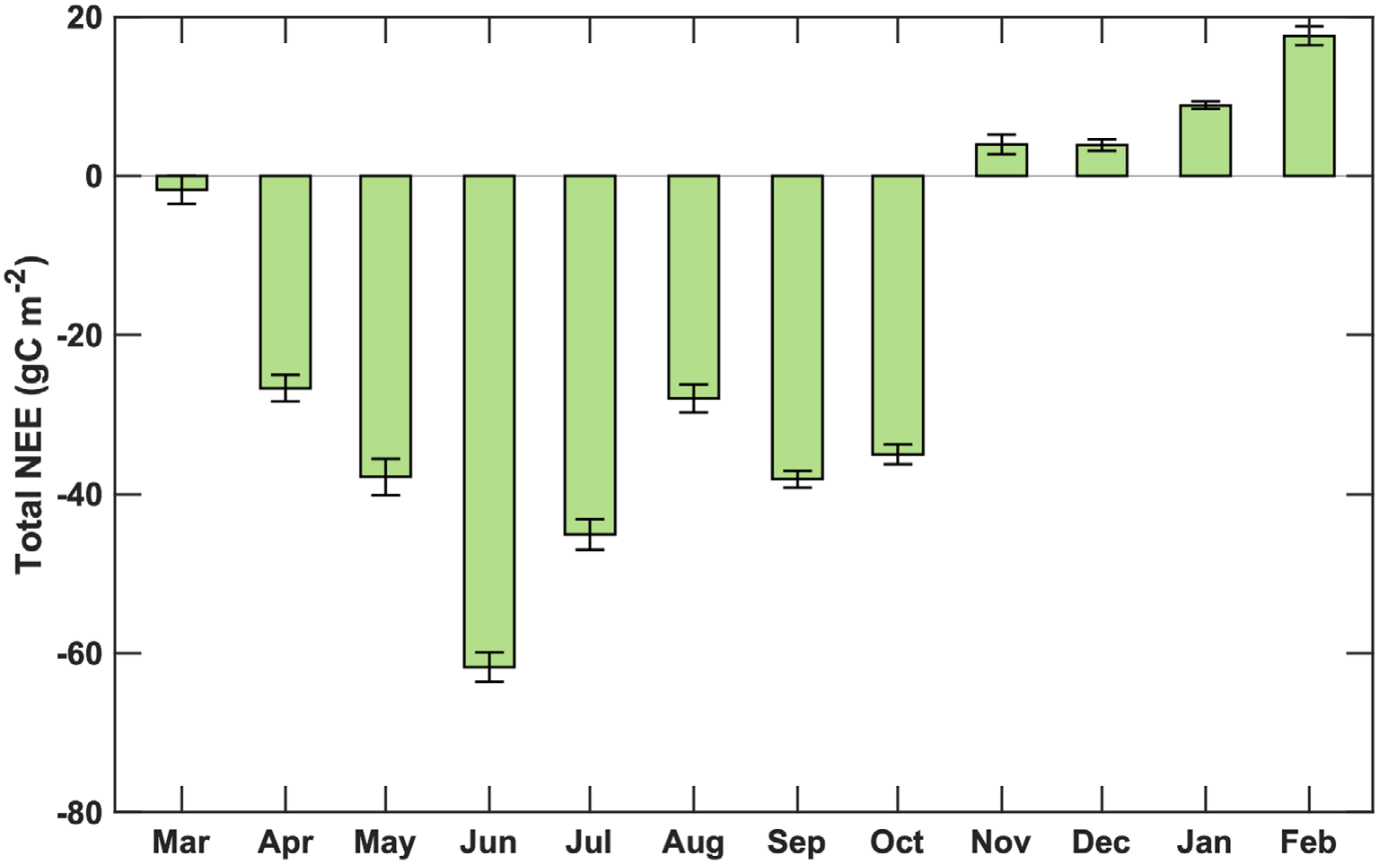
Monthly total net ecosystem exchange (NEE) values obtained for the salt marsh ecosystem during March 2016 to February 2017. During March 2016 to February 2017, the salt marsh exchanged −269.0 ± 9.1 g of carbon per m^2^.

Half‐hourly, gap‐filled NEE values were converted to carbon equivalent and summed over 24‐h periods to obtain daily totals, which were subsequently integrated to derive monthly NEE. Monthly variations reflected changes in biophysical drivers and the amount of physiologically active biomass contributing to CO_2_ exchange above and within the water column. At the onset of the growing season (March to April), monthly integrated NEE averaged −3.2 ± 1.8 g C m^−2^ (Figure 12), coinciding with early Spartina emergence (Figure 3). As biomass accumulated through the peak growing season (June–July), NEE reached −50 to −60 ± 2.3 g C m^−2^ per month. Following canopy senescence, the NEE declined and by November the marsh transitioned to a weak source of carbon (Figure 13). From November through February, the ecosystem remained a net carbon source, with maximum monthly emissions of 20 ± 2.1 g C. Over the full 2016 growing season, the cumulative NEE was −269.1 ± 9.1 g C m^−2^, consistent with annual values reported for other salt marshes (164 g C to 295 g C m^−2^; Yan et al. 2008; Artigas et al. 2015; Forbrich and Giblin 2015; Han et al. 2015). Although a fraction of plant litter decomposes locally and contributes to soil organic matter, approximately 80% of the detrital carbon is exported laterally to adjacent lagoon and ocean subsystems as dissolved inorganic and organic carbon (Wang and Cai 2004; Wang et al. 2016; Najjar et al. 2018).

## 4 Discussion

This study demonstrates a strong seasonal coupling between environmental drivers and vegetation phenology that governs both the magnitude and direction of NEE, with important implications for salt‐marsh carbon cycling under climate change and sea‐level rise. Consistent with observations from other Atlantic and Gulf Coast marshes (Kathilankal et al. 2008; Moffett et al. 2010; Forbrich and Giblin 2015; Knox et al. 2018; Mayen et al. 2024), *Spartina*‐dominated systems function as strong CO_2_ sinks during periods of peak biomass. The annual NEE of −269.1 ± 9.1 g C m^−2^ places the VCR marsh among the upper range of reported salt‐marsh carbon sinks (Yan et al. 2008; Artigas et al. 2015; Han et al. 2015; Nahrawi et al. 2020).

The strength and persistence of this carbon sink are constrained by temperature, radiation amount and quality, and hydrologic forcing, which are expected to shift under future climate scenarios. Temperature exerted a dual control on NEE by simultaneously enhancing photosynthesis and ecosystem respiration, yielding a nonlinear response with an optimum near 303 K. This temperature optimum is consistent with previous site‐level observations (Kathilankal et al. 2011) and broader syntheses of ecosystem carbon fluxes (Baldocchi 2008; Piao et al. 2008; Mahecha et al. 2010). Projected warming is therefore likely to push marsh ecosystems beyond this optimal range more frequently, increasing respiratory losses relative to gross primary production and potentially reducing net carbon uptake or shifting marshes toward carbon neutrality during extreme heat events (Kirwan and Megonigal 2013; Neubauer and Megonigal 2015). Enhanced CO_2_ uptake under diffuse light conditions highlights the importance of radiation quality in regulating marsh productivity. Diffuse radiation improves within‐canopy light distribution and reduces photoinhibition, consistent with the cloud‐enhancement effect observed across diverse ecosystems (Gu, Fuentes, et al. 1999; Gu, Shugart, et al. 1999; Knohl and Baldocchi 2008; Oliphant and Stoy 2018; Hemes et al. 2020). Despite the relatively short canopy and moderate LAI at the VCR marsh, diffuse radiation increased NEE by approximately 30%, suggesting that future changes in cloud regimes and aerosol loading over coastal regions (Painemal et al. 2021; Corral et al. 2021) may exert a first‐order control on marsh carbon uptake. Tidal inundation emerged as a dominant regulator of both daytime and nighttime CO_2_ exchange by reducing effective photosynthetic leaf area, constraining gas diffusion, and limiting oxygen availability for respiration. The observed 30%–60% reduction in NEE is consistent with previous studies of frequently flooded marshes and tidal wetlands (Kathilankal et al. 2008; Han et al. 2015; Nahrawi et al. 2020). As sea‐level rise increases flooding frequency, duration, and depth, marsh carbon uptake is likely to decline unless vertical sediment accretion and vegetation productivity keep pace (Morris et al. 2002; Rogers et al. 2019; Morris and Whiting 2024). Although suppressed respiration during inundation can partially offset reduced photosynthesis, prolonged or chronic flooding is expected to erode net carbon gains. Atmospheric CO_2_ uptake represents only one component of marsh carbon sequestration, as dissolved and particulate carbon are also exported laterally through tidal creeks to adjacent estuarine and coastal water where they can influence aquatic productivity and carbon cycling (Wang and Cai 2004; Najjar et al. 2018). While some studies have documented large lateral carbon exports through direct measurements of dissolved carbon flows, others have found smaller contributions when comparing annual marsh‐atmosphere carbon exchange with longer‐term carbon burial (Forbrich et al. 2018). Salt marshes therefore function both as atmospheric carbon sinks and as integral components of coastal carbon transport. Projected increases in inundation associated with relative sea‐level rise are likely to suppress both photosynthesis and respiration, potentially reducing net CO_2_ uptake and long‐term sequestration capacity, particularly where landward migration is constrained (Kirwan and Megonigal 2013). Increased salinity may further limit net CO_2_ uptake in salt marshes (Alber et al. 2008; Rolando et al. 2023; Russell et al. 2023). A recent study (Mast and Yang 2025) found a midday depression of salt marsh GPP that was best predicted by daily maximum tidal height and air temperature. Supporting this interpretation, Mast and Yang (2025) reported a pronounced midday depression in salt‐marsh gross primary productivity that was best predicted by daily maximum tidal height and air temperature. While the inundation effect is consistent with the present study, the strongest midday suppression of GPP occurred during lower high tides under warm conditions, underscoring the complex interactions among hydrology, interstitial salinity, temperature, and plant physiology. Collectively, these interacting controls highlight the sensitivity of marsh carbon dynamics to climate change and emphasize the need for integrated, process‐based assessments when evaluating the long‐term role of coastal wetlands in climate mitigation.

This study addressed three key questions governing marsh–atmosphere CO_2_ exchange across the full growing season: (1) the effect of light quality on quantum use efficiency, (2) the influence of tidal inundation on CO_2_ fluxes, and (3) the role of environmental drivers in influencing seasonal and annual NEE variability. Despite its low‐stature, sparse canopy (mean height 0.63 ± 0.02 m; LAI 1.5 ± 0.5 m^2^ m^−2^), the marsh exhibited a pronounced enhancement in photosynthetic efficiency under diffuse light. Diffuse light conditions enhanced ecosystem quantum use efficiency threefold (*α*_Cloudy_ = 0.012 ± 0.004 versus *α*_Clear_ = 0.004 ± 0.001 mol CO_2_ per (mol photons)) and raised the photo‐saturation point, yielding ~30% greater CO_2_ assimilation at comparable PAR levels than under clear‐sky conditions. Transient tidal flooding strongly modulated NEE magnitude and variability, with reductions increasing exponentially with inundation depth and peaking at 6.0 ± 0.8 μmol CO_2_ m^−2^ s^−1^ during full canopy submergence (> 0.7 m) in summer. The marsh was inundated during 12% of tidal cycles and submerged 7% of daytime, reducing annual NEE by ~33%. NEE also varied nonlinearly with temperature, increasing to an optimum near 303 K before declining, consistent with enzyme kinetics and respiratory constraints. Integrating these effects, the marsh functioned as a net annual CO_2_ sink, assimilating 269 ± 9 g C m^−2^ between March 2016 and February 2017. Collectively, these findings demonstrate that light quality, temperature, and tidal hydrology jointly regulate the carbon balance of salt marsh ecosystems. The VCR‐LTER marsh provides a valuable reference for anticipating how coastal wetlands will likely respond to ongoing sea‐level rise, warming, and shifts in cloudiness and aerosol loading. Long‐term, ecosystem‐scale flux observations such as these are essential for improving process‐based models of coastal carbon cycling under changing climate and sea‐level regimes.

## Author Contributions

**Jesus Ruiz‐Plancarte:** conceptualization, data curation, formal analysis, funding acquisition, investigation, methodology, software, visualization, writing – original draft, writing – review and editing. **Jose D. Fuentes:** conceptualization, formal analysis, funding acquisition, investigation, methodology, project administration, resources, supervision, writing – review and editing. **Karen J. McGlathery:** conceptualization, funding acquisition, investigation, project administration, resources, supervision, writing – review and editing.

## Funding

This work was supported by the National Science Foundation, Grant numbers 1832221, 2425178, and DGE1255832.

## Conflicts of Interest

The authors declare no conflicts of interest.

## Data Availability

Data sets included in this study are archived at the Ameriflux data archive (https://ameriflux.lbl.gov/sites/siteinfo/US‐VFP/). Data sets included in this article also reside on the VCR LTER data banks (https://www.vcrlter.virginia.edu/home2/?page_id=105).

## References

[gcb70740-bib-0001] Adam, P. 2002. “Salt Marshes in a Time of Change.” Environmental Conservation 29: 39–61. 10.1017/S0376892902000048.

[gcb70740-bib-0002] Alados, I. , and L. Alados‐Arboledas . 1999. “Direct and Diffuse Photosynthetically Active Radiation: Measurements and Modelling.” Agricultural and Forest Meteorology 93: 27–38. 10.1016/S01681923(98)00107-5.

[gcb70740-bib-0003] Alber, M. , E. M. Swenson , S. C. Adamowicz , and I. A. Mendelssohn . 2008. “Salt Marsh Dieback: An Overview of Recent Events in the US.” Estuarine, Coastal and Shelf Science 80: 1–11. 10.1016/j.ecss.2008.08.009.

[gcb70740-bib-0004] Artigas, F. , J. Y. Shin , C. Hobble , A. Marti‐Donati , K. V. R. Schäfer , and I. Pechmann . 2015. “Long Term Carbon Storage Potential and CO_2_ Sink Strength of a Restored Salt Marsh in New Jersey.” Agricultural and Forest Meteorology 200: 313–321. 10.1016/j.agrformet.2014.09.012.

[gcb70740-bib-0005] Baldocchi, D. 2008. “‘Breathing’ of the Terrestrial Biosphere: Lessons Learned From a Global Network of Carbon Dioxide Flux Measurement Systems.” Australian Journal of Botany 56: 1. 10.1071/BT07151.

[gcb70740-bib-0006] Barr, J. G. , V. Engel , J. D. Fuentes , et al. 2010. “Controls on Mangrove Forest‐Atmosphere Carbon Dioxide Exchanges in Western Everglades National Park.” Journal of Geophysical Research – Biogeosciences 115: 1–14. 10.1029/2009JG001186.

[gcb70740-bib-0007] Barr, J. G. , J. D. Fuentes , V. Engel , and J. C. Zieman . 2009. “Physiological Responses of Red Mangroves to the Climate in the Florida Everglades.” Journal of Geophysical Research – Biogeosciences 114: 1–13. 10.1029/2008JG000843.

[gcb70740-bib-0008] Bortolus, A. , P. Adam , J. B. Adams , et al. 2019. “Supporting Spartina: Interdisciplinary Perspective Shows Spartina as a Distinct Solid Genus.” Ecology 100: 1–6. 10.1002/ecy.2863. 31398280

[gcb70740-bib-0009] Cai, W. J. 2011. “Estuarine and Coastal Ocean Carbon Paradox: CO_2_ Sinks or Sites of Terrestrial Carbon Incineration?” Annual Review of Marine Science 3: 123–145. 10.1146/annurev-marine120709-142723. 21329201

[gcb70740-bib-0010] Campbell, G. S. , and J. M. Norman . 1998. An Introduction to Environmental Biophysics. 2nd ed. Springer Science and Business Media. 10.1007/978-1-4612-1626-1.

[gcb70740-bib-0011] Chapin, F. S. , G. M. Woodwell , J. T. Randerson , et al. 2006. “Reconciling Carbon‐Cycle Concepts, Terminology, and Methods.” Ecosystems 9: 1041–1050. 10.1007/s10021-005-0105-7.

[gcb70740-bib-0012] Chen, D. X. , M. Coughenour , A. Knapp , and C. Owensby . 1994. “Mathematical Simulation of C_4_ Grass Photosynthesis in Ambient and Elevated CO_2_ .” Ecological Modelling 73: 63–80. 10.1016/03043800(94)90098-1.

[gcb70740-bib-0013] Chen, H. , and H. Q. Tian . 2005. “Does a General Temperature‐Dependent Q_10_ Model of Soil Respiration Exist at Biome and Global Scale?” Journal of Integrative Plant Biology 47: 1288–1302. 10.1111/j.1744-7909.2005.00211.x.

[gcb70740-bib-0014] Chmura, G. L. 2003. “Global Carbon Sequestration in Tidal, Saline Wetland Soils.” Global Biogeochemical Cycles 17: 1–12. 10.1029/2002GB001917.

[gcb70740-bib-0015] Colmer, T. D. , A. Winkel , and O. Pedersen . 2011. “A Perspective on Underwater Photosynthesis in Submerged Terrestrial Wetland Plants.” AoB Plants 11: 1–15. 10.1093/aobpla/plr030. PMC3249690 22476500

[gcb70740-bib-0016] Corral, A. F. , R. A. Braun , B. Cairns , et al. 2021. “An Overview of Atmospheric Features Over the Western North Atlantic Ocean and North American East Coast–Part 1: Analysis of Aerosols, Gases, and Wet Deposition Chemistry.” Journal of Geophysical Research: Atmospheres 126: e2020JD032592. 10.1029/2020JD032592. PMC8243758 34211820

[gcb70740-bib-0017] Craft, C. 2007. “Freshwater Input Structures Soil Properties, Vertical Accretion, and Nutrient Accumulation of Georgia and U.S. Tidal Marshes.” Limnology and Oceanography 52: 1220–1230. 10.4319/lo.2007.52.3.1220.

[gcb70740-bib-0018] Czapla, K. M. , I. C. Anderson , and C. A. Currin . 2020. “Net Ecosystem Carbon Balance in a North Carolina, USA, Salt Marsh. Journal of Geophysical Research.” Biogeosciences 125: e2019JG005509. 10.1029/2019JG005238.

[gcb70740-bib-0019] Drake, K. , H. Halifax , S. C. Adamowicz , and C. Craft . 2015. “Carbon Sequestration in Tidal Salt Marshes of the Northeast United States.” Environmental Management 56: 998–1008. 10.1007/s00267-015-0568-z. 26108413

[gcb70740-bib-0020] Fagherazzi, S. , and A. M. Priestas . 2010. “Sediments and Water Fluxes in a Muddy Coastline: Interplay Between Waves and Tidal Channel Hydrodynamics.” Earth Surface Processes and Landforms 35: 284–293. 10.1002/esp.1909.

[gcb70740-bib-0021] Fagherazzi, S. , P. L. Wiberg , S. Temmerman , E. Struyf , Y. Zhao , and P. A. Raymond . 2013. “Fluxes of Water, Sediments, and Biogeochemical Compounds in Salt Marshes.” Ecological Processes 2: 1–16. 10.1186/2192-1709-2-3.

[gcb70740-bib-0022] Farquhar, G. D. , S. von Caemmerer , and J. A. Berry . 1980. “A Biochemical Model of Photosynthetic CO_2_ Assimilation in Leaves of C_3_ Species.” Planta 149: 78–90. 10.1007/BF00386231. 24306196

[gcb70740-bib-0023] Fitzgerald, D. M. , and Z. Hughes . 2019. “Marsh Processes and Their Response to Climate Change and Sea‐Level Rise.” Annual Review of Earth and Planetary Sciences 47: 481–517. 10.1146/annurev-earth-082517-010255.

[gcb70740-bib-0024] Forbrich, I. , and A. E. Giblin . 2015. “Marsh‐Atmosphere CO_2_ Exchange in a New England Salt Marsh.” Journal of Geophysical Research: Biogeosciences 120: 1825–1838. 10.1002/2015JG003044.

[gcb70740-bib-0025] Forbrich, I. , A. E. Giblin , and C. S. Hopkinson . 2018. “Constraining Marsh Carbon Budgets Using Long‐Term C Burial and Contemporary Atmospheric CO_2_ Fluxes.” Journal of Geophysical Research: Biogeosciences 123: 867–878. 10.1002/2017JG004336.

[gcb70740-bib-0026] Fratini, G. , and M. Mauder . 2014. “Towards a Consistent Eddy‐Covariance Processing: An Intercomparison of EddyPro and TK3.” Atmospheric Measurement Techniques 7: 2273–2281. 10.5194/amt7-2273-2014.

[gcb70740-bib-0027] Giordano, J. C. P. , M. J. Brush , and I. C. Anderson . 2011. “Quantifying Annual Nitrogen Loads to Virginia's Coastal Lagoons: Sources and Water Quality Response.” Estuaries and Coasts Estuaries and Coasts 34: 297–309. 10.1007/s12237-010-9345-7.

[gcb70740-bib-0028] Gu, L. , D. Baldocchi , S. B. Verma , et al. 2002. “Advantages of Diffuse Radiation for Terrestrial Ecosystem Productivity.” Journal of Geophysical Research: Atmospheres 107: 2. 10.1029/2001JD001242.

[gcb70740-bib-0029] Gu, L. , J. D. Fuentes , H. H. Shugart , R. M. Staebler , and T. A. Black . 1999. “Responses of Net Ecosystem Exchanges of Carbon Dioxide to Changes in Cloudiness: Results From Two North American Deciduous Forests.” Journal of Geophysical Research: Atmospheres 104: 31421. 10.1029/1999JD901068.

[gcb70740-bib-0030] Gu, L. , H. H. Shugart , J. D. Fuentes , T. A. Black , and S. R. Shewchuk . 1999. “Micrometeorology, Biophysical Exchanges and NEE Decomposition in a Two‐Story Boreal Forest ‐ Development and Test of an Integrated Model.” Agricultural and Forest Meteorology 94: 123–148. 10.1016/S0168-1923(99)00006-4.

[gcb70740-bib-0031] Guo, H. , A. Noormets , B. Zhao , et al. 2009. “Tidal Effects on Net Ecosystem Exchange of Carbon in an Estuarine Wetland.” Agricultural and Forest Meteorology 149: 1820–1828. 10.1016/j.agrformet.2009.06.010.

[gcb70740-bib-0032] Han, G. , X. Chu , Q. Xing , et al. 2015. “Effects of Episodic Flooding on the Net Ecosystem CO_2_ Exchange of a Supratidal Wetland in the Yellow River Delta.” Journal of Geophysical Research: Biogeosciences 120: 1506–1520. 10.1002/2017JG003800.

[gcb70740-bib-0033] Hawman, P. A. , D. R. Mishra , J. L. O'Connell , D. L. Cotten , C. R. Narron , and L. Mao . 2021. “Salt Marsh Light Use Efficiency Is Driven by Environmental Gradients and Species‐Specific Physiology and Morphology. Journal of Geophysical Research.” Biogeosciences 126: e2020JG006213. 10.1029/2020JG006213.

[gcb70740-bib-0034] Hemes, K. S. , J. Verfaillie , and D. D. Baldocchi . 2020. “Wildfire‐Smoke Aerosols Lead to Increased Light Use Efficiency Among Agricultural and Restored Wetland Land Uses in California's Central Valley. Journal of Geophysical Research.” Biogeosciences 125: e2019JG005380. 10.1029/2019JG005380.

[gcb70740-bib-0035] Henderson‐Sellers, A. 1989. “North American Total Cloud Amount Variations This Century.” Global and Planetary Change 1: 175–194. 10.1016/0921-8181(89)90002-7.

[gcb70740-bib-0036] Herrmann, M. , R. G. Najjar , W. M. Kemp , et al. 2015. “Net Ecosystem Production and Organic Carbon Balance of U.S. East Coast Estuaries: A Synthesis Approach.” Global Biogeochemical Cycles 29: 96–111. 10.1002/2013GB004736.

[gcb70740-bib-0037] Idaszkin, Y. L. , and A. Bortolus . 2011. “Does Low Temperature Prevent *Spartina alterniflora* From Expanding Toward the Austral‐Most Salt Marshes?” Plant Ecology 212: 553–561. 10.1007/s11258-010-9844-4.

[gcb70740-bib-0038] Ji, F. , Z. Wu , J. Huang , and E. P. Chassignet . 2014. “Evolution of Land Surface Air Temperature Trend.” Nature Climate Change 4: 462–466. 10.1038/nclimate2223.

[gcb70740-bib-0039] Kastler, J. A. , and P. L. Wiberg . 1996. “Sedimentation and Boundary Changes of Virginia Salt Marshes.” Estuarine, Coastal and Shelf Science 42: 683–700. 10.1006/ecss.1996.0044.

[gcb70740-bib-0040] Kathilankal, J. C. , T. J. Mozdzer , J. D. Fuentes , P. D'Odorico , K. J. McGlathery , and J. C. Zieman . 2008. “Tidal Influences on Carbon Assimilation by a Salt Marsh.” Environmental Research Letters 3: 044010. 10.1088/1748-9326/3/4/044010.

[gcb70740-bib-0041] Kathilankal, J. C. , T. J. Mozdzer , J. D. Fuentes , K. J. McGlathery , P. D'Odorico , and J. C. Zieman . 2011. “Physiological Responses of *Spartina alterniflora* to Varying Environmental Conditions in Virginia Marshes.” Hydrobiologia 669: 167–181. 10.1007/s10750-011-0681-9.

[gcb70740-bib-0042] Kirwan, M. L. , and L. K. Blum . 2011. “Enhanced Decomposition Offsets Enhanced Productivity and Soil Carbon Accumulation in Coastal Wetlands Responding to Climate Change.” Biogeosciences 8: 987–993. 10.5194/bg-8-987-2011.

[gcb70740-bib-0043] Kirwan, M. L. , G. R. Guntenspergen , A. D'Alpaos , J. T. Morris , S. M. Mudd , and S. Temmerman . 2010. “Limits on the Adaptability of Coastal Marshes to Rising Sea Level.” Geophysical Research Letters 37: 1–5. 10.1029/2010GL045489.

[gcb70740-bib-0044] Kirwan, M. L. , and J. P. Megonigal . 2013. “Tidal Wetland Stability in the Face of Human Impacts and Sea‐Level Rise.” Nature 504: 53–60. 10.1038/nature12856. 24305148

[gcb70740-bib-0045] Kirwan, M. L. , J. P. Megonigal , G. L. Noyce , and A. J. Smith . 2023. “Geomorphic and Ecological Constraints on the Coastal Carbon Sink.” Nature Reviews Earth and Environment 4: 393–406.

[gcb70740-bib-0046] Kirwan, M. L. , and S. M. Mudd . 2012. “Response of Salt‐Marsh Carbon Accumulation to Climate Change.” Nature 489: 550–553. 10.1038/nature11440. 23018965

[gcb70740-bib-0047] Kljun, N. , P. Calanca , M. W. Rotach , and H. P. Schmid . 2015. “A Simple Two‐Dimensional Parameterisation for Flux Footprint Prediction (FFP).” Geoscientific Model Development 8: 3695–3713. 10.5194/gmd-8-3695-2015.

[gcb70740-bib-0048] Knohl, A. , and D. D. Baldocchi . 2008. “Effects of Diffuse Radiation on Canopy Gas Exchange Processes in a Forest Ecosystem.” Journal of Geophysical Research 113: G02023. 10.1029/2007JG000663.

[gcb70740-bib-0049] Knox, S. H. , L. Windham‐Myers , F. Anderson , C. Sturtevant , and B. Bergamaschi . 2018. “Direct and Indirect Effects of Tides on Ecosystem‐Scale CO_2_ Exchange in a Brackish Tidal Marsh in Northern California.” Journal of Geophysical Research: Biogeosciences 123: 787–806. 10.1002/2017JG004048.

[gcb70740-bib-0050] Koebsch, F. , S. Glatzel , J. Hofmann , I. Forbrich , and G. Jurasinski . 2013. “CO_2_ Exchange of a Temperate Fen During the Conversion From Moderately Rewetting to Flooding.” Journal of Geophysical Research: Biogeosciences 118: 940–950. 10.1002/jgrg.20069.

[gcb70740-bib-0051] Lasslop, G. , M. Reichstein , D. Papale , et al. 2010. “Separation of Net Ecosystem Exchange Into Assimilation and Respiration Using a Light Response Curve Approach: Critical Issues and Global Evaluation.” Global Change Biology 16: 187–208. 10.1111/j.1365-2486.2009.02041.x.

[gcb70740-bib-0052] Law, B. E. , E. Falge , L. Gu , et al. 2002. “Environmental Controls Over Carbon Dioxide and Water Vapor Exchange of Terrestrial Vegetation.” Agriculture and Forest Meteorology 113: 97–120. 10.1016/S0168-1923(02)00104-1.

[gcb70740-bib-0053] Lee, M. S. , D. Y. Hollinger , T. F. Keenan , A. P. Ouimette , S. V. Ollinger , and A. D. Richardson . 2018. “Model‐Based Analysis of the Impact of Diffuse Radiation on CO_2_ Exchange in a Temperate Deciduous Forest.” Agricultural and Forest Meteorology 249: 377–389. 10.1016/j.agrformet.2017.11.016.

[gcb70740-bib-0054] Lee, S.‐C. , C.‐J. Fan , Z.‐Y. Wu , and J.‐Y. Juang . 2015. “Investigating Effect of Environmental Controls on Dynamics of CO_2_ Budget in a Subtropical Estuarial Marsh Wetland Ecosystem.” Environmental Research Letters 10: 025005. 10.1088/1748-9326/10/2/025005.

[gcb70740-bib-0055] Lloyd, J. , and J. A. Taylor . 1994. “On the Temperature Dependence of Soil Respiration.” Functional Ecology 8: 315–323. 10.2307/2389824.

[gcb70740-bib-0056] Loomis, M. J. , and C. B. Craft . 2010. “Carbon Sequestration and Nutrient (Nitrogen, Phosphorus) Accumulation in River‐Dominated Tidal Marshes, Georgia, USA.” Soil Science Society of America Journal 74: 1028. 10.2136/sssaj2009.0171.

[gcb70740-bib-0057] Macreadie, P. I. , A. R. Hughes , and D. L. Kimbro . 2013. “Loss of “Blue Carbon” From Coastal Salt Marshes Following Habitat Disturbance.” PLoS One 8: 1–8. 10.1371/journal.pone.0069244. PMC3704532 23861964

[gcb70740-bib-0058] Mahecha, M. D. , M. Reichstein , N. Carvalhais , et al. 2010. “Global Convergence in the Temperature Sensitivity of Respiration at Ecosystem Level.” Science 329: 838–840. 10.1126/science.1189587. 20603495

[gcb70740-bib-0059] Malone, S. L. , J. Barr , J. D. Fuentes , et al. 2016. “Sensitivity to Low‐Temperature Events: Implications for CO_2_ Dynamics in Subtropical Coastal Ecosystems.” Wetlands 36: 957–967. 10.1007/s13157-016-0810-3.

[gcb70740-bib-0060] Mariotti, G. , and J. Carr . 2014. “Dual Role of Salt Marsh Retreat: Long‐Term Loss and Short‐Term Resilience.” Water Resources Research 50: 2963–2974. 10.1002/2013WR014676.

[gcb70740-bib-0061] Mast, H. M. , and X. Yang . 2025. “Midday Depression of Photosynthesis in *Spartina alterniflora* in a Virginia Salt Marsh. Journal of Geophysical Research.” Biogeosciences 130: e2024JG008338. 10.1029/2024JG008338.

[gcb70740-bib-0500] Mayen, J. , P. Polsenaere , É. Lamaud , et al. 2024. “Atmospheric CO_2_ Exchanges Measured by eddy Covariance Over a Temperate Salt Marsh and Influence of Environmental Controlling Factors.” Biogeosciences 21: 993–1016. 10.5194/bg-21-993-2024.

[gcb70740-bib-0062] McGlathery, K. J. , K. Sundbäck , and I. C. Anderson . 2007. “Eutrophication in Shallow Coastal Bays and Lagoons: The Role of Plants in the Coastal Filter.” Marine Ecology Progress Series 348: 1–18. 10.3354/meps07132.

[gcb70740-bib-0063] Mcleod, E. , G. L. Chmura , S. Bouillon , et al. 2011. “A Blueprint for Blue Carbon: Toward an Improved Understanding of the Role of Vegetated Coastal Habitats in Sequestering CO_2_ .” Frontiers in Ecology and the Environment 9: 552–560. 10.1890/110004.

[gcb70740-bib-0064] McNicol, G. , and W. L. Silver . 2014. “Separate Effects of Flooding and Anaerobiosis on Soil Greenhouse Gas Emissions and Redox Sensitive Biogeochemistry.” Journal of Geophysical Research: Biogeosciences 119: 1129–1146. 10.1002/2013JG002569.

[gcb70740-bib-0065] Mitsch, W. J. , and J. G. Gosselink . 2015. Wetlands. John Wiley & Sons, Inc.

[gcb70740-bib-0066] Moffat, A. M. , D. Papale , M. Reichstein , et al. 2007. “Comprehensive Comparison of Gap‐Filling Techniques for Eddy Covariance Net Carbon Fluxes.” Agricultural and Forest Meteorology 147: 209–232. 10.1016/j.agrformet.2007.08.011.

[gcb70740-bib-0067] Moffett, K. B. , A. Wolf , J. A. Berry , and S. M. Gorelick . 2010. “Salt Marsh‐Atmosphere Exchange of Energy, Water Vapor, and Carbon Dioxide: Effects of Tidal Flooding and Biophysical Controls.” Water Resources Research 46: W10525. 10.1029/2009WR009041.

[gcb70740-bib-0068] Morris, J. T. , B. Haskin , and J. T. Morris . 1990. “A 5‐Yr Record of Aerial Primary Production and Stand Characteristics of *Spartina alterniflora* .” Ecology 71: 2209–2217. 10.2307/1938633.

[gcb70740-bib-0069] Morris, J. T. , P. V. Sundareshwar , C. T. Nietch , B. Kjerfve , and D. R. Cahoon . 2002. “Responses of Coastal Wetlands to Rising Sea Level.” Ecology 83: 2869–2877. 10.1890/0012-9658(2002)083[2869:ROCWTR]2.0.CO;2.

[gcb70740-bib-0070] Morris, J. T. , and G. J. Whiting . 2024. “Components of the CO_2_ Exchange in a Southeastern USA Salt Marsh.” Ocean‐Land‐Atmosphere Research 3: 0077. 10.34133/olar.0077.

[gcb70740-bib-0071] Mudd, S. M. , A. D'Alpaos , and J. T. Morris . 2010. “How Does Vegetation Affect Sedimentation on Tidal Marshes? Investigating Particle Capture and Hydrodynamic Controls on Biologically Mediated Sedimentation.” Journal of Geophysical Research: Earth Surface 115: 1–14. 10.1029/2009JF001566.

[gcb70740-bib-0072] Nahrawi, H. , M. Y. Leclerc , S. Pennings , G. Zhang , N. Singh , and R. Pahari . 2020. “Impact of Tidal Inundation on the Net Ecosystem Exchange in Daytime Conditions in a Salt Marsh.” Agricultural and Forest Meteorology 294: 108133. 10.1016/j.agrformet.2020.108133.

[gcb70740-bib-0073] Najjar, R. G. , M. Heermann , R. Alexander , et al. 2018. “Carbon Budget of Tidal Wetlands, Estuaries, and Shelf Waters of Eastern North America.” Global Biogeochemical Cycles 32: 389–416. 10.1002/2017GB005790.

[gcb70740-bib-0074] Neubauer, S. C. , and J. P. Megonigal . 2015. “Moving Beyond Global Warming Potentials to Quantify the Climatic Role of Ecosystems.” Ecosystems 18: 1000–1013. 10.1007/s10021-015-9879-4.

[gcb70740-bib-0075] Oliphant, A. J. , and P. C. Stoy . 2018. “An Evaluation of Semi‐Empirical Models for Partitioning Photosynthetically Active Radiation Into Diffuse and Direct Beam Components.” Journal of Geophysical Research: Biogeosciences 123: 889–901. 10.1002/2017JG004370.

[gcb70740-bib-0076] Oliveira, P. J. C. , E. L. Davin , S. Levis , and S. I. Seneviratne . 2011. “Vegetation‐Mediated Impacts of Trends in Global Radiation on Land Hydrology: A Global Sensitivity Study.” Global Change Biology 17: 3453–3467. 10.1111/j.1365-2486.2011.02506.x.

[gcb70740-bib-0077] Painemal, D. , A. F. Corral , A. Sorooshian , et al. 2021. “An Overview of Atmospheric Features Over the Western North Atlantic Ocean and North American East Coast—Part 2: Circulation, Boundary Layer, and Clouds.” Journal of Geophysical Research: Atmospheres 126: e2020JD033423. 10.1029/2020JD033423. PMC8243758 34211820

[gcb70740-bib-0078] Peng, S. , S. Piao , T. Wang , J. Sun , and Z. Shen . 2009. “Temperature Sensitivity of Soil Respiration in Different Ecosystems in China.” Soil Biology and Biochemistry 41: 1008–1014. 10.1016/j.soilbio.2008.10.023.

[gcb70740-bib-0079] Penman, H. L. 1948. “Natural Evaporation From Open Water, Bare Soil and Grass.” Proceedings of the Royal Society of London. Series A: Mathematical and Physical Sciences 193: 120–145. 10.1098/rspa.1948.0037. 18865817

[gcb70740-bib-0080] Perez, R. , P. Ineichen , R. Seals , J. Michalsky , and R. Stewart . 1990. “Modeling Daylight Availability and Irradiance Components From Direct and Global Irradiance.” Solar Energy 44: 271–289. 10.1016/0038-092X(90)90055-H.

[gcb70740-bib-0081] Peterson, P. M. , K. Romaschenko , Y. H. Arrieta , and J. M. Saarela . 2014a. “A Molecular Phylogeny and New Subgeneric Classification of Sporobolus (Poaceae: Chloridoideae: Sporobolinae).” Taxon 63: 1212–1243. 10.12705/636.19.

[gcb70740-bib-0082] Peterson, P. M. , K. Romaschenko , Y. H. Arrieta , and J. M. Saarela . 2014b. “Proposal to Conserve the Name Sporobolus Against Spartina, Crypsis, Ponceletia, and Heleochloa (Poaceae: Chloridoideae: Sporobolinae).” Taxon 63: 1373–1374. 10.12705/636.23.

[gcb70740-bib-0083] Piao, S. , P. Ciais , P. Friedlingstein , et al. 2008. “Net Carbon Dioxide Losses of Northern Ecosystems in Response to Autumn Warming.” Nature 451: 49–52. 10.1038/nature06444. 18172494

[gcb70740-bib-0085] Reagan, J. R. , D. Seidov , Z. Wang , et al. 2024. World Ocean Atlas 2023, Volume 2: Salinity. NOAA National Centers for Environmental Information (NCEI Accession 0270533). Vol. 90. NOAA Atlas NESDIS. 10.25923/70qt-9574.

[gcb70740-bib-0086] Regnier, P. , L. Resplandy , R. G. Najjar , and P. Ciais . 2022. “The Land‐To‐Ocean Loops of the Global Carbon Cycle.” Nature 603: 401–410. 10.1038/s41586-021-04339-9. 35296840

[gcb70740-bib-0087] Reichstein, M. , E. Falge , D. Baldocchi , et al. 2005. “On the Separation of Net Ecosystem Exchange Into Assimilation and Ecosystem Respiration: Review and Improved Algorithm.” Global Change Biology 11: 1424–1439. 10.1111/j.1365-2486.2005.001002.x.

[gcb70740-bib-0088] Reindl, D. T. , W. A. Beckman , and J. A. Duffie . 1990. “Diffuse Fraction Correlations.” Solar Energy 45: 1–7. 10.1016/0038-092X(90)90060-P.

[gcb70740-bib-0089] Roderick, M. L. , G. D. Farquhar , S. L. Berry , and I. R. Noble . 2001. “On the Direct Effect of Clouds and Atmospheric Particles on the Productivity and Structure of Vegetation.” Oecologia 129: 21–30. 10.1007/s004420100760. 28547064

[gcb70740-bib-0090] Rogers, K. , J. J. Kelleway , N. Saintilan , et al. 2019. “Wetland Carbon Storage Controlled by Millennial‐Scale Variation in Relative Sea‐Level Rise.” Nature 567: 91–95. 10.1038/s41586-019-0951-7. 30842636

[gcb70740-bib-0091] Rolando, J. L. , M. Hodges , K. D. Garcia , et al. 2023. “Restoration and Resilience to Sea Level Rise of a Salt Marsh Affected by Dieback Events.” Ecosphere 14: e4467. 10.1002/ecs2.4467.

[gcb70740-bib-0092] Ruimy, A. , P. G. Jarvis , D. D. Baldocchi , and B. Saugier . 1995. “CO_2_ Fluxes Over Plant Canopies and Solar Radiation: A Review.” Advances in Ecological Research 26, no. C: 1–68. 10.1016/S0065-2504(08)60063-X.

[gcb70740-bib-0093] Russell, S. J. , L. Windham‐Myers , E. J. Stuart‐Haentjens , et al. 2023. “Increased Salinity Decreases Annual Gross Primary Productivity at a Northern California Brackish Tidal Marsh.” Environmental Research Letters 18: 34045. 10.1088/1748-9326/acbbdf.

[gcb70740-bib-0094] Ryu, Y. , C. Jiang , H. Kobayashi , and M. Detto . 2018. “MODIS‐Derived Global Land Products of Shortwave Radiation and Diffuse and Total Photosynthetically Active Radiation at 5 Km Resolution From 2000.” Remote Sensing of Environment 204: 812–825. 10.1016/j.rse.2017.09.021.

[gcb70740-bib-0095] Sallenger, A. H. , K. S. Doran , and P. A. Howd . 2012. “Hotspot of Accelerated Sea‐Level Rise on the Atlantic Coast of North America.” Nature Climate Change 2: 884–888. 10.1038/NCLIMATE1597.

[gcb70740-bib-0096] Schäfer, K. , R. Tripathee , F. Artigas , T. H. Morin , and G. Bohrer . 2014. “Carbon Dioxide Fluxes of an Urban Tidal Marsh in the Hudson‐Raritan Estuary.” Journal of Geophysical Research: Biogeosciences 119: 703–721. 10.1002/2013JG002522.Received.

[gcb70740-bib-0097] Schedlbauer, J. L. , S. F. Oberbauer , G. Starr , and K. L. Jimenez . 2010. “Seasonal Differences in the CO2 Exchange of a Short‐Hydroperiod Florida Everglades Marsh.” Agricultural and Forest Meteorology 150: 994–1006. 10.1016/j.agrformet.2010.03.005.

[gcb70740-bib-0098] Smalley, A. 1960. “Energy Flow of a Salt Marsh Grasshopper Population.” Ecology 41: 672–677. 10.1017/CBO9781107415324.004.

[gcb70740-bib-0099] Spitters, C. J. T. , H. A. J. M. Toussaint , and J. Goudriaan . 1986. “Separating the Diffuse and Direct Component of Global Radiation and Its Implications for Modeling Canopy Photosynthesis Part I. Components of Incoming Radiation.” Agricultural and Forest Meteorology 38: 217–229. 10.1016/0168-1923(86)90060-2.

[gcb70740-bib-0100] Tanner, C. B. , and G. W. Thurtell . 1969. Anemoclinometer Measurements of Reynolds Stress and Heat Transport in the Atmospheric Surface Layer (No. ECOM66G22F).

[gcb70740-bib-0101] Teal, J. M. , and B. L. Howes . 1996. “Interannual Variability of a Salt‐Marsh Ecosystem.” Limnology and Oceanography 41: 802–809. 10.4319/lo.1996.41.4.0802.

[gcb70740-bib-0102] Troxler, T. G. , J. G. Barr , J. D. Fuentes , et al. 2015. “Component‐Specific Dynamics of Riverine Mangrove CO_2_ Efflux in the Florida Coastal Everglades.” Agricultural and Forest Meteorology 213: 273–282. 10.1016/j.agrformet.2014.12.012.

[gcb70740-bib-0103] Turner, D. P. , W. D. Ritts , J. M. Styles , et al. 2006. “A Diagnostic Carbon Flux Model to Monitor the Effects of Disturbance and Interannual Variation in Climate on Regional NEP.” Tellus Series B: Chemical and Physical Meteorology 58: 476–490. 10.1111/j.1600-0889.2006.00221.x.

[gcb70740-bib-0104] Vasquez, E. A. , E. P. Glenn , G. R. Guntenspergen , J. J. Brown , and S. G. Nelson . 2006. “Salt Tolerance and Osmotic Adjustment of *Spartina alterniflora* (Poaceae) and the Invasive M Haplotype of *Phragmites australis* (Poaceae) Along a Salinity Gradient.” American Journal of Botany 93: 1784–1790. 10.3732/ajb.93.12.1784. 21642124

[gcb70740-bib-0105] Vázquez‐Lule, A. , and R. Vargas . 2021. “Biophysical Drivers of Net Ecosystem and Methane Exchange Across Phenological Phases in a Tidal Salt Marsh.” Agricultural and Forest Meteorology 300: 108309. 10.1016/j.agrformet.2020.108309.

[gcb70740-bib-0106] Vickers, D. , and L. Mahrt . 1997. “Quality Control and Flux Sampling Problems for Tower and Aircraft Data.” Journal of Atmospheric and Oceanic Technology 14: 512–526.

[gcb70740-bib-0107] Wang, F. , X. Lu , C. J. Sanders , and J. Tang . 2019. “Tidal Wetland Resilience to Sea Level Rise Increases Their Carbon Sequestration Capacity in United States.” Nature Communications 10: 1–11. 10.1038/s41467-019-13294-z. PMC6883032 31780651

[gcb70740-bib-0108] Wang, Z. A. , and W.‐J. Cai . 2004. “Carbon Dioxide Degassing and Inorganic Carbon Export From a Marsh‐Dominated Estuary (The Duplin River): A Marsh CO_2_ Pump.” Limnology and Oceanography 49: 341–354. 10.4319/lo.2004.49.2.0341. PMC5812098 29456267

[gcb70740-bib-0109] Wang, Z. A. , K. D. Kroeger , N. K. Ganju , M. E. Gonneea , and S. N. Chu . 2016. “Intertidal Salt Marshes as an Important Source of Inorganic Carbon to the Coastal Ocean.” Limnology and Oceanography 2: 1916–1931. 10.1002/lno.10347.

[gcb70740-bib-0110] Webb, E. K. , G. I. Pearman , and R. Leuning . 1980. “Correction of Flux Measurements for Density Effects due to Heat and Water Vapour Transfer.” Quarterly Journal of the Royal Meteorological Society 106: 85–100. 10.1002/qj.49710644707.

[gcb70740-bib-0111] Wilczak, J. M. , S. P. Oncley , and S. A. Stage . 2001. “Sonic Anemometer Tilt Correction Algorithms.” Boundary‐Layer Meteorology 99: 127–150. 10.1023/A:1018966204465.

[gcb70740-bib-0112] Wohlfahrt, G. , A. Hammerle , A. Haslwanter , M. Bahn , U. Tappeiner , and A. Cernusca . 2008. “Disentangling Leaf Area and Environmental Effects on the Response of the Net Ecosystem CO_2_ Exchange to Diffuse Radiation.” Geophysical Research Letters 35: 1–5. 10.1029/2008GL035090. PMC3858830 24347740

[gcb70740-bib-0113] Wu, Z. , P. Dijkstra , G. W. Koch , J. Peñuelas , and B. A. Hungate . 2011. “Responses of Terrestrial Ecosystems to Temperature and Precipitation Change: A Meta‐Analysis of Experimental Manipulation.” Global Change Biology 17: 927–942. 10.1111/j.1365-2486.2010.02302.x.

[gcb70740-bib-0114] Yan, Y. , B. Zhao , J. Chen , et al. 2008. “Closing the Carbon Budget of Estuarine Wetlands With Tower‐Based Measurements and MODIS Time Series.” Global Change Biology 14: 1690–1702. 10.1111/j.1365-2486.2008.01589.x.

[gcb70740-bib-0115] Yuan, W. , W. Cai , J. Xia , et al. 2014. “Global Comparison of Light Use Efficiency Models for Simulating Terrestrial Vegetation Gross Primary Production Based on the LaThuile Database.” Agricultural and Forest Meteorology 192: 108–120. 10.1016/j.agrformet.2014.03.007.

[gcb70740-bib-0116] Zhong, Q. , K. Wang , Q. Lai , C. Zhang , L. Zheng , and J. Wang . 2016. “Carbon Dioxide Fluxes and Their Environmental Control in a Reclaimed Coastal Wetland in the Yangtze Estuary.” Estuaries and Coasts 39: 344–362. 10.1007/s12237-015-9997-4.

